# Single-cell transcriptomic atlas of blood and lung from mice infected with SARS-CoV-2 revealing distinct virulence characteristics between prototype and Omicron BA.1 strain

**DOI:** 10.1080/21505594.2025.2548931

**Published:** 2025-08-28

**Authors:** Na Rong, Jiaying Yao, Hui Quan, Jing Wu, Binbin Zhao, Wanjun Peng, Hekai Yang, Gengxin Zhang, Xiaoyue Ding, Xiaohui Wei, Jiangning Liu

**Affiliations:** a NHC Key Laboratory of Human Disease Comparative Medicine, State Key Laboratory of Respiratory Health and Multimorbidity, Institute of Laboratory Animal Science, Chinese Academy of Medical Sciences and Comparative Medicine Center, Peking Union Medical College, Beijing, China; b Annoroad Gene Technology (Beijing) Co., Ltd, Beijing, China; c Servicebio, Wuhan, China

**Keywords:** SARS-CoV-2 prototype strain, Omicron BA.1 variant, pathogenicity differences, single-cell transcriptome, neutrophil, monocyte-macrophage

## Abstract

The markedly reduced pathogenicity of Severe Acute Respiratory Syndrome Coronavirus 2 (SARS-CoV-2) Omicron variant in comparison to earlier strains has raised critical questions regarding its underlying mechanisms. To elucidate the host immune responses driving these differences, we performed single-cell transcriptomic profiling of lung and blood samples from human angiotensin-converting enzyme 2 (hACE2) transgenic mice infected with either the SARS-CoV-2 prototype strain or the Omicron BA.1 variant at 5 days post-inoculation. Both strains induced a reduction in lung cell numbers, with capillary endothelial cells showing the highest number of differentially expressed genes (DEGs). Shared transcriptional responses included upregulation of chemokine (e.g. *Gnaq*, *Lyn*, *Ccl5*) and IL-17 signaling pathways. Notably, Omicron BA.1 infection resulted in downregulation of *Txnip*, a key gene involved in oxidative stress responses. Genes associated with neutrophil granules and pro-inflammatory functions (*Mmp8*, *S100a8*, *S100a9*) were also downregulated, whereas wound healing pathways were upregulated in immature neutrophils. Additionally, Omicron BA.1 altered gene expression associated with neutrophil migration from blood to lung, and decreased the activation of cell chemotaxis, cytokine-mediated signaling, IL-17 and NF-κB pathways in pro-inflammatory monocytes and pulmonary interstitial macrophages. These findings highlight distinct immunological signatures contributing to the attenuated pathogenicity of Omicron BA.1, particularly through the modulation of neutrophil and monocyte-macrophage responses.

## Graphical abstract

**Figure:**
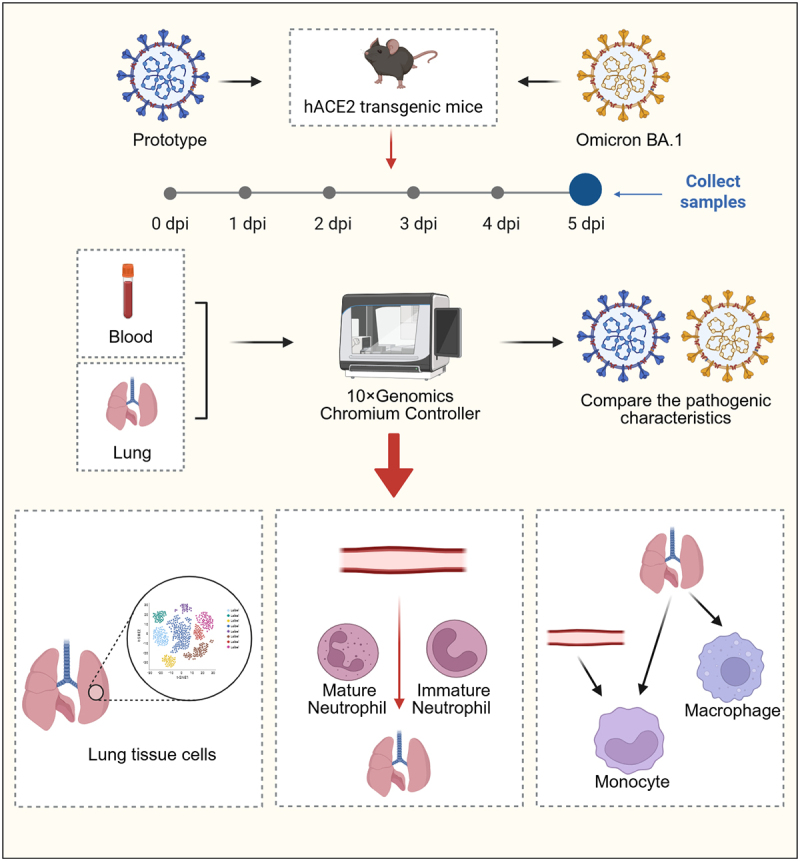

## Introduction

Severe Acute Respiratory Syndrome Coronavirus 2 (SARS-CoV-2) has become a significant global health concern since its initial emergence in 2019. The virus has been observed to manifest a diverse range of clinical presentations, including asymptomatic, mild, and even more severe forms such as pneumonia or acute respiratory distress syndrome (ARDS) [1,2]. Because of its capacity for constant mutation, the Omicron variant has rapidly become dominant. Clinical data demonstrate that Omicron infections, while highly transmissible, are associated with diminished lower respiratory tract involvement and severe outcomes, primarily manifesting as upper respiratory symptoms [3–5]. Notably, the BA.1 sublineage represented the initial and dramatic global surge of Omicron, fundamentally altering the pandemic landscape by establishing its attenuated phenotype. While subsequent subvariants (e.g. BA.2, BA.4, BA.5) also exhibit reduced pathogenicity, BA.1 serves as the pivotal strain for comparing the mechanisms underlying this significant attenuation against the highly pathogenic prototype strain. To further elucidate the pathogenicity characteristics of the different strains, a substantial number of animal experiments have been conducted to study the differences in pathogenicity between Omicron and prototype strains. Consistent with clinical observations, studies in key animal models (transgenic mice, hamsters, non-human primates) have demonstrated significantly reduced pathogenicity for Omicron BA.1, including attenuated weight loss, lower pulmonary viral loads, and less severe lung pathology compared to prototype infection [4,6–8]. In our previous study, we similarly demonstrated that the Omicron BA.1 variant resulted in disease alleviation compared to the prototype strain in a hACE2 transgenic mouse model [7]. Endothelial cell dysfunction and inflammatory cell accumulation have been identified as key features of severe cases of Corona Virus Disease 2019 (COVID-19), ultimately leading to pulmonary endothelial thrombosis [9]. However, there is a paucity of comprehensive studies that directly compare the cellular-level pathogenesis of lung tissue cells in Omicron BA.1 and prototype strains.

The innate immune response plays a pivotal role in determining the course of the initial SARS-CoV-2 infection. Type I interferon response (IFN-I), cytokine storm, and myeloid compartment dysregulation are significant contributors to inflammatory dysregulation in SARS-CoV-2 infection [10–12]. Neutrophils, which represent the initial line of defense against microbial pathogens in the human body, exhibit a substantial degree of infiltration in patients with COVID-19 [13]. The activation of neutrophils and increased neutrophil extracellular traps (NETs) production have emerged as prognostic indicators of unfavorable outcomes [14]. Furthermore, immature neutrophils that emerge in patients with SARS-CoV-2 infection have been examined using flow cytometry and single-cell RNA sequencing (scRNA-seq), revealing altered functional phenotypes [15–17]. Monocytes and macrophages have been repeatedly proposed as pivotal mediators of the hyperinflammatory state in patients with SARS-CoV-2 infection [18]. Several studies have documented the accumulation of specific monocyte and macrophage subsets in individuals with SARS-CoV-2 infection [19, 20]. scRNA-seq has also been used to study the transformation and transcriptional signature of different phenotypes of monocytes and macrophages in infectious diseases [21]. However, comparative analyses of these critical innate immune populations, particularly neutrophils and monocyte-macrophage dynamics, between prototype and Omicron BA.1 infections at a detailed cellular and transcriptional level are lacking. The gene expression trajectories of neutrophils migrating from blood to lung tissue also remain poorly defined.

scRNA-seq has emerged as a powerful tool in clinical and fundamental research on SARS-CoV-2. Notwithstanding the pervasiveness of scRNA-seq in the analysis of peripheral blood and tissue samples from fatal cases in previous literature [22], the declining prevalence of the prototype strain and the relatively low lethality of Omicron BA.1 present significant challenges in obtaining samples from patients infected with these strains, particularly tissue samples. Additionally, scRNA-seq was employed to examine the alterations in immune responses induced by SARS-CoV-2 and the factors influencing its susceptibility in peripheral blood and lung tissues from non-human primates, mice, and hamsters [23–25]. However, the majority of these studies concentrated on the evaluation of pharmaceutical agents or the examination of a singular strain in animals of varying ages. Therefore, this study employs scRNA-seq to systematically compare the immune landscape and cellular injury in the blood and lungs during infection with the SARS-CoV-2 prototype strain versus the Omicron BA.1 variant. We specifically focus on defining the differential impact on lung tissue cells, and key innate immune effectors (neutrophils, monocytes, macrophages), aiming to elucidate the cellular and molecular basis underlying attenuated pathogenicity of Omicron BA.1.

## Materials and methods

### Ethic statements

PGK-hACE2 humanized mice were raised and infected in an Animal Biosafety Level 3 (ABSL-3) facility. The mouse experiments were approved and overseen by the Institutional Animal Care and Use Committee of the Institute of Laboratory Animal Science, Chinese Academy of Medical Sciences & Peking Union Medical College (LJN22007 and LJN23014). All procedures were performed in accordance with the relevant ethical norms.

### Mouse experiments

Specific pathogen-free (SPF) PGK-hACE2 humanized mice (6–8 weeks old) from the Beijing HFK Bioscience Co., Ltd and Institute of Zoology, Chinese Academy of Sciences were used for the infectious experiment. SARS-CoV-2 prototype (SARS-CoV-2/WH-09/human/2020/CHN; GenBank: MT093631.2) and Omicron BA. 1 (GenBank: OM095411.1) were provided by the Institute of Laboratory Animal Science, Chinese Academy of Medical Sciences & Peking Union Medical College. We used hACE2 mice, inoculated with the SARS-CoV-2 prototype and Omicron BA.1 strain, respectively, and conducted a daily observation of the changes in body weight and mortality in each group. As demonstrated in our preceding studies, 5 days post-inoculation (dpi) is a pivotal time point primarily employed for temporal analyses of interactions and post-infection outcomes, with specific emphasis on pathogenicity and pathological distinctions between prototype and BA.1 infections [9]. Consequently, we procured peripheral blood and lung tissues from mice at 5 days post inoculation (dpi) for pathological testing as well as single cell transcriptome analysis. The mice used in the above experiments were nasally inoculated with 50 μL of SARS-CoV-2 prototype or Omicron BA.1 at a dose of 1 × 10^5^ TCID_50_ after anesthesia, and the negative control (NC) group was established by inoculation of 50 μL phosphate buffer saline (PBS). In the neutrophil and macrophage depletion experiments, mice were challenged with 50 μL of SARS-CoV-2 prototype at a dose of 1 × 10^3^ TCID_50_. Three groups of mice (*n* = 16 per group) were intraperitoneally injected with 200 μL PBS, 400 μg of anti-Ly6G antibody diluted with 200 µL (Bio X Cell, USA), and 200 μL clodronate liposomes (Yeasen, China) every other day from the challenge day, respectively, until 10 dpi. Body weights, clinical symptoms and mortality were daily recorded.

### RNA extraction and quantitative RT-PCR

Total RNA was extracted from the blood and lungs using the TRIzol (Thermo Fisher Scientific, USA) method. Quantitative Probe RT-PCR kit (Qiagen, Germany) was used to perform quantitative real-time PCR according to the manufacturer’s instructions. Standard curves were generated from a series of 10-fold dilutions of recombinant plasmids harboring N gene fragment of SARS-CoV-2 at known concentrations. The primer sequence of SARS-CoV-2 based on the nucleocapsid protein (N) gene was as follows: probe: FAM-TTGCTGCTGCTTGACAGATT-TAM; forward primer, 5′-GGGGAACTTCTCCTGCTAGAAT-3′; reverse primer, 5′- CAGACATTTTGCTCTCAAGCTG-3′. qRT-PCR reactions were conducted with the following cycling protocol and primer treatment: 50°C for 30 min, 95°C for 15 min, then followed by 45 cycles at 95°C for 15s and 60°C for 1 min.

### Pathological examination

Lung tissues were fixed in formalin and processed into 4 μm thick paraffin sections. The sections were then stained with hematoxylin and eosin (H&E) and observed under a light microscope.

### Single-cell RNA-seq sequencing

Blood and lung samples (Prototype group, *n* = 3; Omicron BA.1 group, *n* = 4; NC group, *n* = 3) were collected at 5 dpi. After a series of measures including lysis of red blood cells and digestion, single cell suspension was obtained. With the use of a Chromium Single Cell 3’ GEM, Library & Gel Bead Kit v3 (10x Genomics, PN-1000075), the SIC single-cell suspension (700–1200 living cells per ml as determined by the CellDrop FL Cell Counter) was loaded onto a Chromium Single Cell Chip (Chromium Single Cell B Chip Kit, 10x Genomics, PN-1000073) according to the manufacturer’s instructions, for co-encapsulation with barcoded Gel Beads at a target capture rate of 6000 individual cells per sample. We lysed captured cells and released RNA was barcoded through reverse-transcription in individual single-cell gel beads in the emulsion (GEMS). In each droplet, complementary DNA (cDNA) was generated and amplified through reverse-transcription on a T100 PCR Thermal Cycler (Bio Rad) at 53°C for 45 min, followed by 85°C for 5 min and a hold at 4°C. Then, cDNA concentration and quality were assessed using Qubit Fluorometer (Thermo Scientific) and Bioanalyzer 2100 (Agilent), respectively. According to the manufacturer’s instructions, scRNA-seq libraries were constructed and sequenced to a depth of 80,000 reads per cell on a NovaSeq 6000 platform (Illumina).

### Single cell RNA-seq data processing

FASTQ files of 20 samples were processed using Cell Ranger (v.6.1.2) count pipeline coupled with the mouse reference version Mus_musculus. GRCm38.89 to generate feature-barcode matrices, respectively. Firstly, we filtered cells that were predicted as double cells using the scrublet software. Next, Seurat object was generated by Seurat package (v. 4.3.0) [26] with R software (v.4.2.2) following these criteria: (1) min.cells = 3; (2) 200 = < nFeature_RNA ≤10000; (3) percent.mt ≤ 0.2; (4) HB.percent ≤0.05. In other words, genes expressed in at least three cells and cells detected gene number ranging from 200 to 10,000 were kept for further analysis. Low-quality cells were also filtered if > 20% UMIs derived from the mitochondrial genome or > 5% UMIs derived from erythrocyte cells.

### Dimensionality reduction and clustering

To remove batch effects across samples, harmony analysis method was used for data integration [27]. In detail, we initially normalized the filtered gene expression data with NormalizeData function. Then the top 2,000 variable genes identified with FindVariableFeatures function were used for RunPCA. Finally, we used RunHarmony with parameters (group.by.vars = “sample”) and the top 20 dimensions for using RunUMAP. Finally, we clustered cells by using the FindNeighbors and FindClusters (resolution = 0.6) functions. The dimensionality reduction and clustering of the blood and lung tissue cells were performed, respectively. The neutrophils, blood myeloid cells and lung myeloid cells were re-clustered similarly with the above harmony analysis method.

### Cluster marker identification and differentially expressed genes (DEG) identification

The FindAllMarkers function in Seurat was used to find markers for each identified cluster. Clusters were then classified and annotated based on the expressions of canonical markers of particular cell types. FindMarkers function in Seurat was used to identify DEGs between two groups of cells with default parameters (log2fc.threshold = 0.25, test.use= “wilcox,” min.pct = 0.1).

### Gene ontology (GO) and Kyoto encyclopedia of genes and genomes (KEGG)

Differential gene sets were annotated with GO and KEGG database using clusterProfiler package (v.3.15.2) [28]. Enrichment pathways were obtained with p.adjust < 0.05. *p* value adjustment (FDR) was performed using the Benjamini – Hochberg method.

### Gene set variation analysis (GSVA) and gene set enrichment analysis (GSEA) analysis

We collected candidate GO and KEGG from enrichment terms and estimated the variation of gene sets for each cell type using average expression among groups with the GSVA or GSEA package [29].

### Neutrophils maturation score

The maturation score of neutrophils was evaluated using AddModuleScore function from Seurat, the genes of which were obtained from Xie et al. [30].

### Quantification and statistical analysis

The statistical significance of the differences in viral load and body weight change was analyzed using *t* tests. All the values are expressed as mean ± SEM (standard error of the Mean). The significant differences in survival percent were analyzed by log-rank test. Statistical analyses were performed using GraphPad prism. Wilcox.test was used for comparisons of continuous variables of gene expression and cell proportions between two groups. Kolmogorov-Smirnov (KS) test was used for GSEA. Fisher’s exact test was used for comparisons of categorical variables such as GO and KEGG enrichment analysis. *p* value adjustment (FDR) is performed using Benjamini-Hochberg method. All statistical analyses for scRNA-seq data were performed in R (v4.2.0). *p* value < 0.05 was considered significant.

## Results

### The single-cell transcriptome of blood and lung

**Figure 1.**
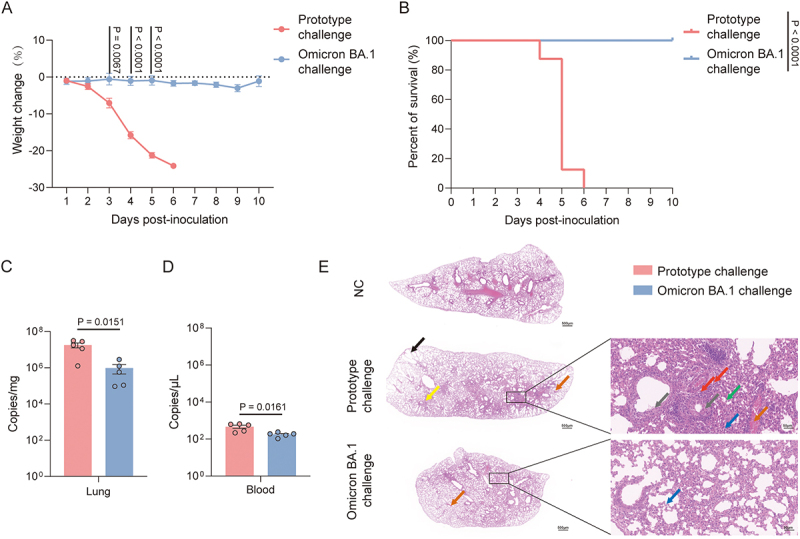
Characteristics of the mouse model challenged with SARS-CoV-2 prototype or Omicron BA.1. (A) Weight change (*n* = 8 in each group). (B) Percent of survival (*n* = 8 in each group). The annotated number is the *p* value. (C-D) Virus RNA quantities in lung and blood of the mice challenged with SARS-CoV-2 prototype or Omicron BA.1 (*n* = 5 in each group) at 5 dpi. (E) Representative H&E results of the lung (The blue arrows point to granulocyte infiltrates; the black arrows point to thickening of the alveolar wall; the green arrows point to macrophages and lymphocytes infiltrates; the gray arrows point to mucosal epithelial cells necrosis; the red arrows point to lymphocytic and granulocytic infiltrates around vessels; the yellow arrows point to perivascular hemorrhage; the orange arrows point to interstitial vascular congestion); scale bars (left: 500 μm; right: 50 μm) are shown.

Prior to scRNA-seq analysis, we conducted an analysis of the phenotypic characteristics of mice infected with the prototype and Omicron BA.1 strains. As observed over time, mice infected with the prototype strain exhibited a notable loss of weight at 4 dpi (Figure 1(A)). By 5 dpi, the majority of mice had lost more than 20% of their initial weight except one, reaching a point where further observation was deemed unethical and the mice were euthanized. However, no significant weight loss was observed in mice infected with the Omicron BA.1 strain (Figure 1(A)). Ultimately, all mice in the prototype group died from the infection by 6 dpi, whereas none of the mice in the Omicron BA.1 group died (Figure 1(B)). The viral RNA in the lungs of the prototype group reached 10^7^ copies/mg at 5 dpi, which was significantly higher than that observed in the Omicron BA.1 group (Figure 1(C)). The viral RNA of peripheral blood (PB) in both groups was below 10^3^ copies/μL at 5 dpi (Figure 1(D)). Furthermore, the lung tissue of the prototype group exhibited more pronounced pathological damage, including increased inflammatory infiltrate composed of neutrophils and macrophages (Figure 1(E)). In our previous study, the proportion of immune cells in mice was examined at various time points after infection with these two strains. It was found that a significant increase in the proportion of neutrophils was observed in the peripheral blood and lung tissues of mice infected with both the strains at 5 dpi. Although no significant changes in the proportion of macrophages have been observed [7]. We further investigated the changes in lung tissue damage caused by the prototype and Omicron BA.1 strain, as well as the changes in immune cells in the blood and lungs at the gene expression level, infected mice at 5 dpi were selected for scRNA-seq analysis.

**Figure 2.**
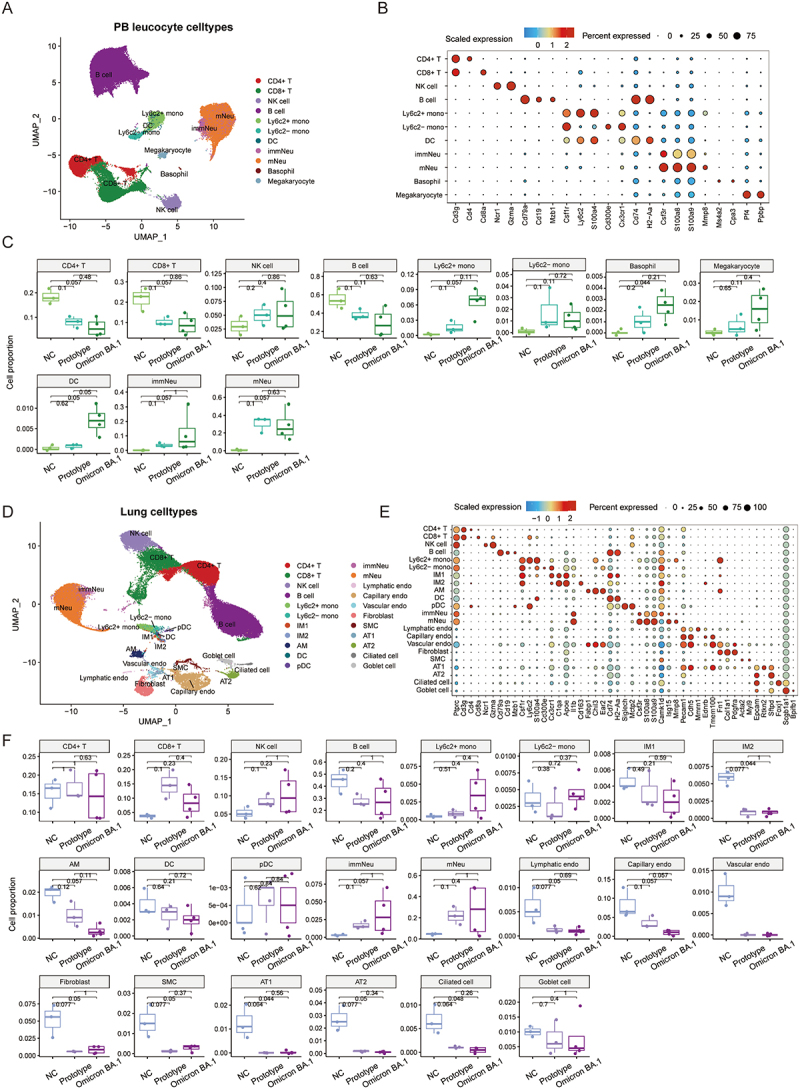
Single cell atlas of blood and lung of mice challenged with SARS-CoV-2 prototype or Omicron BA.1. (A) Uniform manifold approximation and projection (UMAP) analysis of annotated cell types in PB leucocytes. (B) Dot plot of representative marker genes with annotated cell types in PB leucocytes. (C) Boxplot plots of PB leucocyte cell types distribution in NC, prototype or Omicron BA.1 infection conditions, each point represents a sample, the annotated number is the *P* value. (D) UMAP analysis of annotated cell types in lung. (E) Dot plot of representative marker genes with annotated cell types in lung. (F) Boxplot plots of lung cell types distribution in NC, prototype or Omicron BA.1 infection conditions, each point represents a sample, the annotated number is the *P* value.

We performed scRNA-seq analysis of peripheral blood leukocytes and lung samples from mice that had undergone different treatments (Figure S1). A total of 74,538 and 70,058 high-quality single cells were obtained from blood and lungs, respectively. Eleven cell types were manually annotated in PB leucocyte using uniform manifold approximation and projection (UMAP) analysis, including CD4^+^T cells, CD8^+^T cells, natural killer (NK) cells, B cells, Ly6c2^+^ monocytes, Ly6c2^−^monocytes, dendritic cells (DC), immature neutrophils (immNeu), mature neutrophils (mNeu), basophils, and megakaryocytes (Figure 2(A) and S2A). The type-specific markers and the top five expression genes were utilized to distinguish the cell types in the blood, as evidenced by the dot plot and heatmap (Figure 2(B) and S2(B)). The percentage of each cell type was visualized by boxplots, bar plots and Sankey diagrams (Figure 2(C) and S2(C,D)). Compared with the uninfected mice (NC group), CD4^+^T cells and CD8^+^T cells decreased, while the proportion of immature neutrophils, mature neutrophils, NK cells, Ly6c2^+^monocytes, Ly6c2^−^monocytes, DCs, basophils, and megakaryocytes increased in mice infected with SARS-CoV-2 prototype and Omicron BA.1, respectively (Figure 2(C)). The proportion of Ly6c2^+^monocytes, DCs, basophils, and megakaryocytes was higher in Omicron BA.1 group than that in the prototype group.

22 cell types were annotated in the lung tissue, including nine clusters in PB leukocytes, as well as in pDC cells, alveolar macrophages (AM), alveolar type I epithelial cells (AT1), alveolar type II epithelial cells (AT2), capillary endothelial cells, vascular endothelial cells, goblet cells, type I pulmonary interstitial macrophages (IM1), type II pulmonary interstitial macrophages (IM2), lymphatic endothelial cells, smooth muscle cells (SMC), fibroblasts, and ciliated cells (Figure 2(D,E) and S2(E)). The typical gene markers and top 5 genes expressed in each cell type were shown in Figure 2(E) and S1(F). Compared to the NC group, the proportion of CD8^+^T cells, NK cells, mature neutrophils and immature neutrophils in the lung tissue of both infection groups increased. The number of other cells, including AMs, IM1s, IM2s, AT1s, AT2s, capillary endothelial cells, lymphatic endothelial cells, SMC, and ciliated cells, exhibited a downward trend in both infection groups (Figure 2(F) and S2(F-H)).

### Common changes in lung gene expression induced by SARS-CoV-2 prototype and Omicron BA.1

**Figure 3.**
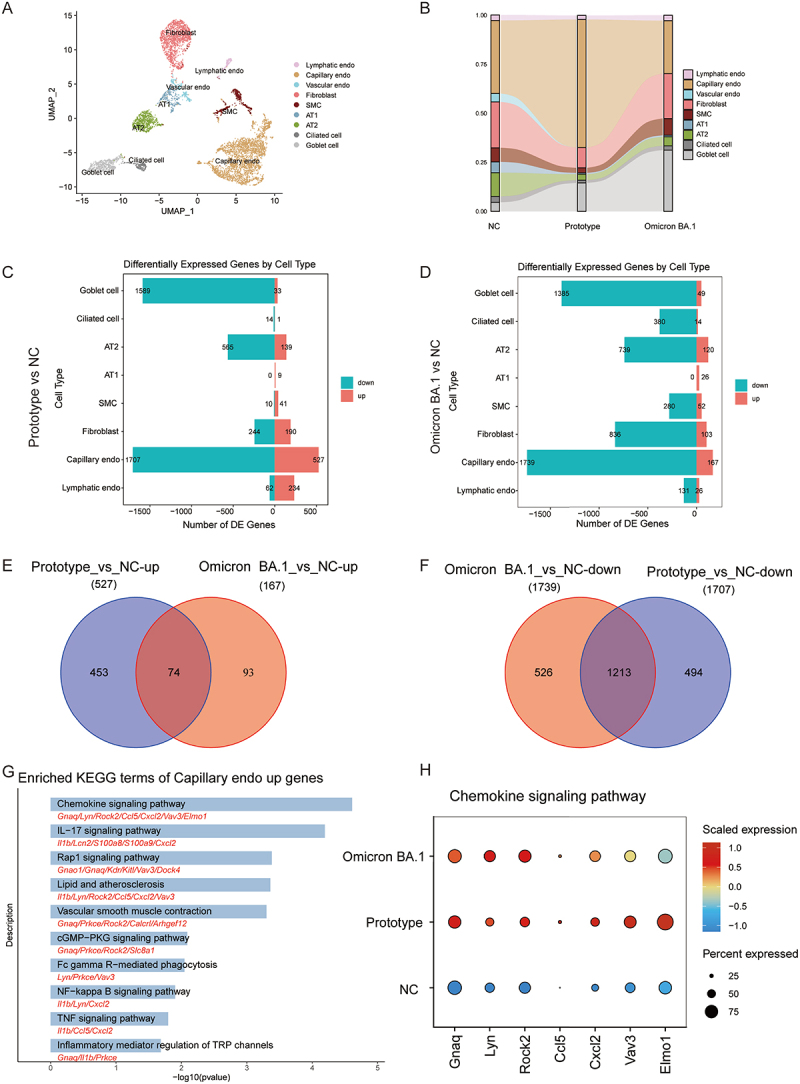
Transcriptional alterations in lung tissue cell types of mice challenged with SARS-CoV-2 prototype or Omicron BA.1. (A) UMAP analysis of lung tissue cells. (B) Sankey diagrams plot of lung cell types distribution in NC, prototype or Omicron BA.1 infection conditions. (C) the up and down DEGs of each cell type in lung of prototype infection vs NC conditions, (|logfc| > 0.25, adjusted *p* value < 0.05). (D) the up and down DEGs of each cell type in Omicron BA.1 infection vs NC conditions (|logfc| > 0.25, adjusted *p* value < 0.05). (E) Venn diagram of up-regulated genes in Capillary_endo, and the numbers in the graph indicate the specific number of DEGs. (F) Venn diagram of down-regulated genes in capillary endo, and the numbers in the graph indicate the specific number of DEGs. (G) KEGG functional enrichment bar plot obtained from 74 co-upregulated genes. (H) Dot plots diagram of the genes enriched in chemokine signaling pathway.

We employed UMAP to perform unsupervised clustering on all lung tissue cells except immune cells, subsequently visualizing the proportion of cell types within the entire lung tissue (Figure 3(A,B) and S3(A)). Identification of numerous DEGs in each cell type in the Prototype vs. NC and Omicron BA.1 vs. NC conditions indicated extensive transcriptomic dysregulation (Figure 3(C,D) and S3(B,C)). It is worthy of note that the highest number of DEGs was observed in capillary endothelial cells in each comparison condition. A total of 527 upregulated and 1,707 downregulated DEGs were identified in capillary endothelial cells when comparing the prototype group with the NC group (Figure 3(C)). In contrast, the capillary endothelial cells in the Omicron BA.1 group demonstrated 167 upregulated and 1,739 downregulated DEGs (Figure 3(D)). To gain further insight into the gene regulation of capillary endothelial cells in response to SARS-CoV-2 infection, we conducted comparative analyses of DEGs in the prototype and Omicron BA.1 groups relative to the NC group. The results revealed that 74 DEGs exhibited co-upregulation, whereas 1,213 DEGs demonstrated co-downregulation (Figure 3(E,F)). Notably, the KEGG and GO terminology annotation indicated that the co-upregulated DEGs were involved in the chemokine signaling, IL-17 signaling, Rap1 signaling, cell migration, and other immune response signaling pathways (Figure 3(G) and S4(A)). Genes related to chemokine signaling pathway, including *Gnaq*, *Lyn*, *Rock2*, *Ccl5*, *Cxcl2*, *Vav3* and *Elmo1*, were found to be upregulated in both groups (Figure 3(H) and S5). The 1,213 genes that were co-downregulated were mainly enriched in ferroptosis, translation, and ribosome-related signaling pathways (Figure S4(B,C)).

### Differences in the effects of prototype and Omicron BA.1 on lung tissue cells

**Figure 4.**
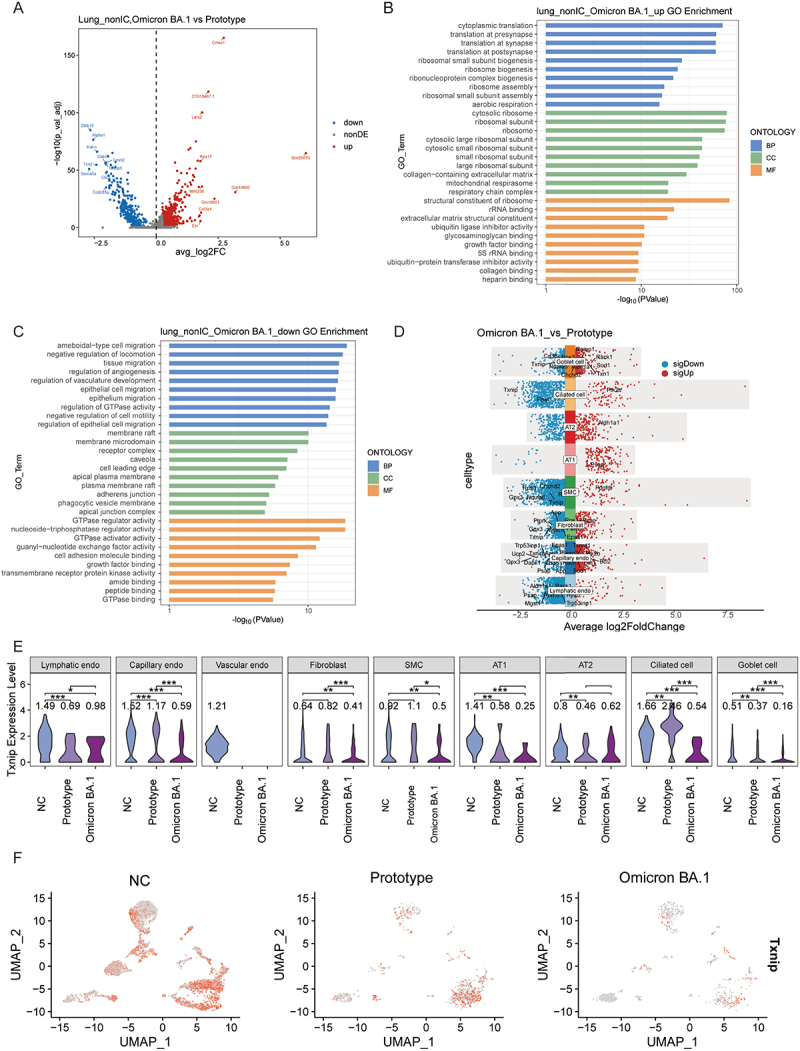
Expression differences between prototype and Omicron BA.1 infected lungs. (A) the volcano plot of DEGs between Omicron BA.1 and prototype (|logfc| > 0.25, adjusted *p* value < 0.05). (B) Bar chart of top 10 GO terms of upregulated DEGs between Omicron BA.1 and prototype in lungs. (C) Bar chart of top 10 GO terms of downregulated DEGs between Omicron BA.1 and prototype in lungs. (D) the volcano plot of up-regulated and down-regulated DEGs in each cell type in Omicron BA.1 vs prototype infection conditions. (E) The violin diagram drawn the expression of *Txnip* gene in annotated cell type group. *: *p* < 0.05; **: *p* < 0.01; ***: *p* < 0.001. (F) The expression of *Txnip* gene in the 3 groups of lung tissue cells UMAP, the degree of gene expression was positively correlated with the degree of redness.

To gain further insight into the reasons why the prototype caused more severe lung injury than BA.1, we identified 808 DEGs in the lungs between the two groups (Figure 4(A)). Subsequently, GO and KEGG analyses were performed on the upregulated and downregulated DEGs to investigate the alterations that occurred in the lung tissues of the two infected groups (Figure 4(B,C), and S6). Genes upregulated in the BA.1 group were predominantly enriched in the signaling pathways of translation and ribosome-related biosynthesis. Conversely, the downregulated genes were mainly enriched in pathways related to the regulation of angiogenesis, epithelial cell migration, cell adhesion, GTPase, and chemokine signaling pathways (Figure 4(B,C) and S6). Subsequently, the expression of the DEGs was examined in each cell type. It was observed that the majority of genes involved in the response to oxidative stress (GO:0006979) were downregulated in the Omicron BA.1 group, particularly in capillary endothelial cells, lymphatic endothelial cells, fibroblasts, SMCs, goblet cells, and ciliated cells (Figure 4(D)). In this analysis, Thioredoxin interacting protein (*Txnip*) gene was downregulated in four cell types, including goblet cells, SMCs, fibroblasts, and capillary endothelial cells, in the Omicron BA.1 group (Figure 4(D)). Therefore, the expression of *Txnip* in various cell types was visualized using a violin diagram, which demonstrated that the expression of *Txnip* in capillary endothelial cells, fibroblasts, SMCs, ciliated cells, and goblet cells was significantly lower in the Omicron BA.1 group than that in the prototype group (Figure 4(E,F)).

### Differential gene expression of neutrophil subpopulations in SARS-CoV-2 prototype and Omicron BA.1

**Figure 5.**
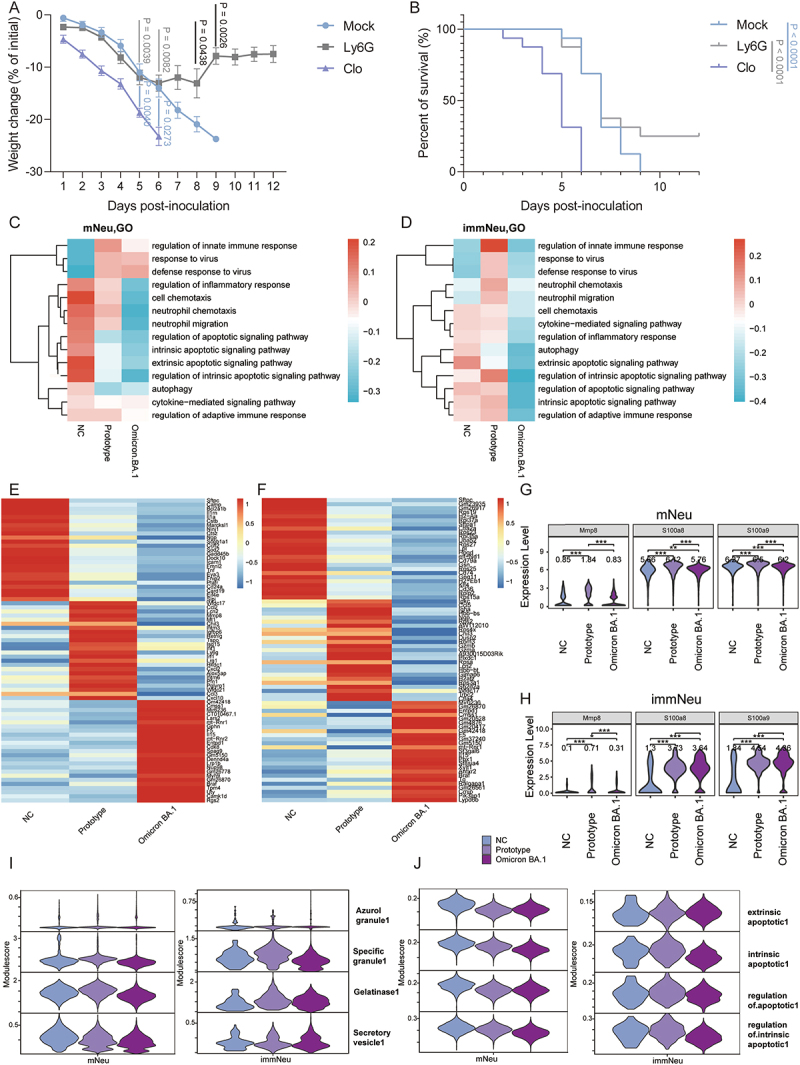
Analysis of neutrophils subsets in lung of prototype and Omicron BA.1 infected mice. A &B. The symptoms of prototype-infected mice after inhibition of neutrophil (Ly6G-treatment) or macrophage (Clo-treatment). (A) Weight change (The *p* values are noted at two time points with the great significant difference between each of two groups). (B) Percent of survival. The uninfected mice were used as mock control. *n* = 16 in each group. The black annotation is the mock vs Ly6G group, the blue annotation is the Mock vs Clo group, and the gray annotation is the Ly6G vs Clo group. (C) Heatmap of GSVA scores with candidate interested GO terms among NC, prototype or Omicron BA.1 infection conditions in mNeu in the lungs. (D) Heatmap of GSVA scores with candidate interested GO terms among NC, prototype or Omicron BA.1 infection conditions in immNeu in the lungs. (E) Heatmaps of top 25 differential genes among NC, prototype or Omicron BA.1 infection conditions in mNeu in the lungs. (F) Heatmaps of top 25 differential genes among NC, prototype or Omicron BA.1 infection conditions with immNeu in the lungs. (G) Expression comparison of *Mmp8*, *S100a8* and *S100a9* genes among NC, prototype and Omicron BA.1 in mNeu of lungs. (H) Expression comparison of *Mmp8*, *S100a8* and *S100a9* genes among NC, prototype and Omicron BA.1 in immNeu of lungs. (I) the violin diagram showed the score of neutrophil granule gene in immNeu and mNeu of lungs. (J) the violin diagram drawn the expression of apoptotic pathways in immNeu and mNeu of lungs. *: *p* < 0.05; **: *p* < 0.01; ***: *p* < 0.001.

As shown previously, the proportion of neutrophils in the blood and lung of mice infected with SARS-CoV-2 prototype or Omicron BA.1 both increases at 5 dpi [7]. The same phenomenon was observed in the annotated immNeu and mNeu in the present study (Figure 2(C,F)). To gain further insight into the biological processes involved in the interaction between neutrophils and SARS-CoV-2, an antibody targeting Ly6G was administered to prototype-infected mice. A reduction in body weight was observed in all the mice at 4 dpi (Figure 5(A,B)). Mice in the Mock group demonstrated a gradual weight loss at 5 dpi, which ultimately resulted in mortality or reached a human endpoint. In contrast, four mice in the Ly6G-treated group exhibited a gradual recovery in body weight (Figure 5(A,B)). GSVA of selected signaling pathways relevant to the function of the neutrophil population in the lungs revealed the upregulation of multiple pathways, including regulating the innate immune response, response to the virus, and defense response to the virus, in mature neutrophils from both infected groups (Figure 5(C)). The GSVA of immNeu revealed that multiple pathways associated with neutrophil function including neutrophil chemotaxis and migration, as well as defense response to virus, exhibited upregulation exclusively in the lungs of the prototype group (Figure 5(D)). Additionally, the top 25 DEGs of mNeu and immNeu in each group were illustrated (Figure 5(E,F)). These findings indicated the activation of immature neutrophils in prototype-infected lungs, in comparison to the Omicron BA.1 strain. The function of immature neutrophils may contribute to a more pronounced inflammatory response in lung tissue infected with the prototype strain. Nevertheless, our findings indicated that there was no evidence of upregulation of the selected functionally relevant signaling pathways in either mature or immature neutrophils in the blood of infected groups (Figure S7A-D). Furthermore, an examination was conducted on the gene expression associated with the key viral clearance mechanisms including neutrophil recruitment and infiltration (e.g. *Mmp8*, *S100a8*, and *S100a9*) and neutrophil degranulation. The expression levels of *Mmp8* were found to be elevated primarily in neutrophil subpopulations within the prototype group. Furthermore, the expression levels of *S100A8* and *S100A9* were significantly elevated in both mNeu and immNeu in the lungs of the infected groups. Overall, the highest expression of *Mmp8*, *S100a8*, and *S100a9* in neutrophil subpopulations was observed in prototype-infected lung tissues (Figure 5(G,H)). The expression of *Mmp8*, *S100a8* and *S100a9* in the mNeu and immNeu of infection groups was predominantly decreased in the blood (Figure S7E and F). The genes encoding specific granules and gelatinase, which are associated with degranulation, exhibited lower levels in the lungs and blood of Omicron BA.1 group than that of the prototype group (Figure 5(I), S7G and S8). This suggests that neutrophils in the prototype-infected group may exhibit enhanced degranulation and killing ability. Furthermore, our findings revealed downregulation of neutrophil apoptosis pathways in Omicron BA.1 infection when compared to the prototype (Figure 5(J) and S7H).

**Figure 6.**
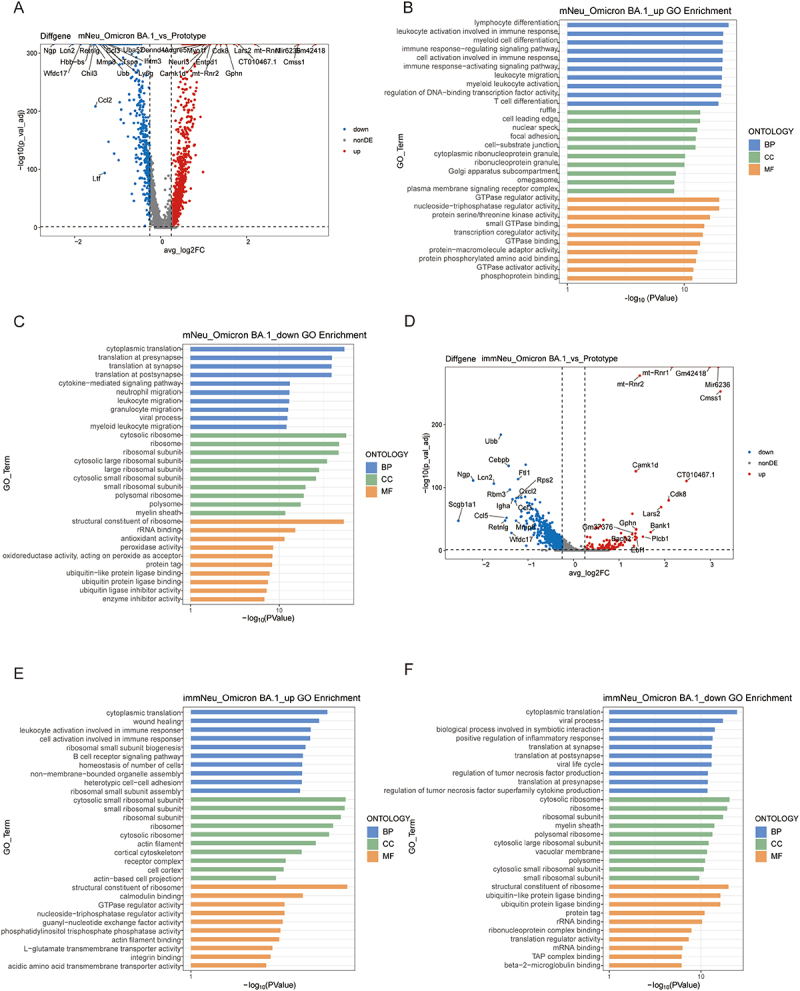
Comparsion of signaling pathways regulation in mNeu and imNeu between prototype and Omicron BA.1 infected lungs. (A) the volcano plot of DEGs in mNeu between prototype and Omicron BA.1 infected lungs (|logfc| > 0.25, adjusted *p* value < 0.05). (B) Bar chart of top 10 GO terms of upregulated DEGs in mNeu between Omicron BA.1 and prototype infected lungs. (C) Bar chart of top 10 GO terms in mNeu of downregulated DEGs between Omicron BA.1 and prototype infected lungs. (D) The volcano plot of DEGs in immNeu between prototype and Omicron BA.1 infected lungs (|logfc| > 0.25, adjusted *p* value < 0.05). (E) Bar chart of top 10 GO terms of upregulated DEGs in immNeu between Omicron BA.1 and prototype infected lungs. (F) Bar chart of top 10 GO terms in immNeu of downregulated DEGs between Omicron BA.1 and prototype infected lungs.

Next, we focused on the differences in the regulation of signaling pathways in the neutrophil subpopulations during infection between the two strains of SARS-CoV-2. A large number of DEGs were obtained by analyzing mNeu from the lung tissues of the Omicron BA.1 and prototype groups. It was found that mNeu downregulated genes related to neutrophil secondary granules such as *Ltf*, *Ngp* and *Lcn2* in the Omicron BA.1 group compared to that in the prototype group (Figure 6(A)). mNeu demonstrated upregulated lymphocyte differentiation, myeloid cell differentiation, immune response regulation, chemokine signaling and pathways associated with cell activation, while exhibiting downregulation of viral process, cytokine-mediated signaling pathway, apoptosis, neutrophil migration and other cell migration pathways in Omicron BA.1 infection (Figure 6(B,C) and S9(A, B)). Additionally, in the case of the Omicron BA.1 group, immNeu also demonstrated downregulation of genes, including *Ngp*, *Lcn2*, and *Mmp8* (Figure 6(D)). Specifically, compared to the prototype group, immNeu in the Omicron BA.1 group demonstrated an upregulation of the wound healing signaling pathway, whereas the positive regulation of the inflammatory response, apoptosis as well as the viral process signaling pathway were downregulated (Figure 6(E,F) and S9(C,D)). This suggests the presence of immature neutrophils in the Omicron BA.1 group is a potential contributor to lung tissue repair, whereas immature neutrophils in the prototype group may be responsible for an inflammatory response that results in further damage to the lung tissue.

### Gene expression alterations in neutrophil subpopulations from the peripheral blood to the lungs

**Figure 7.**
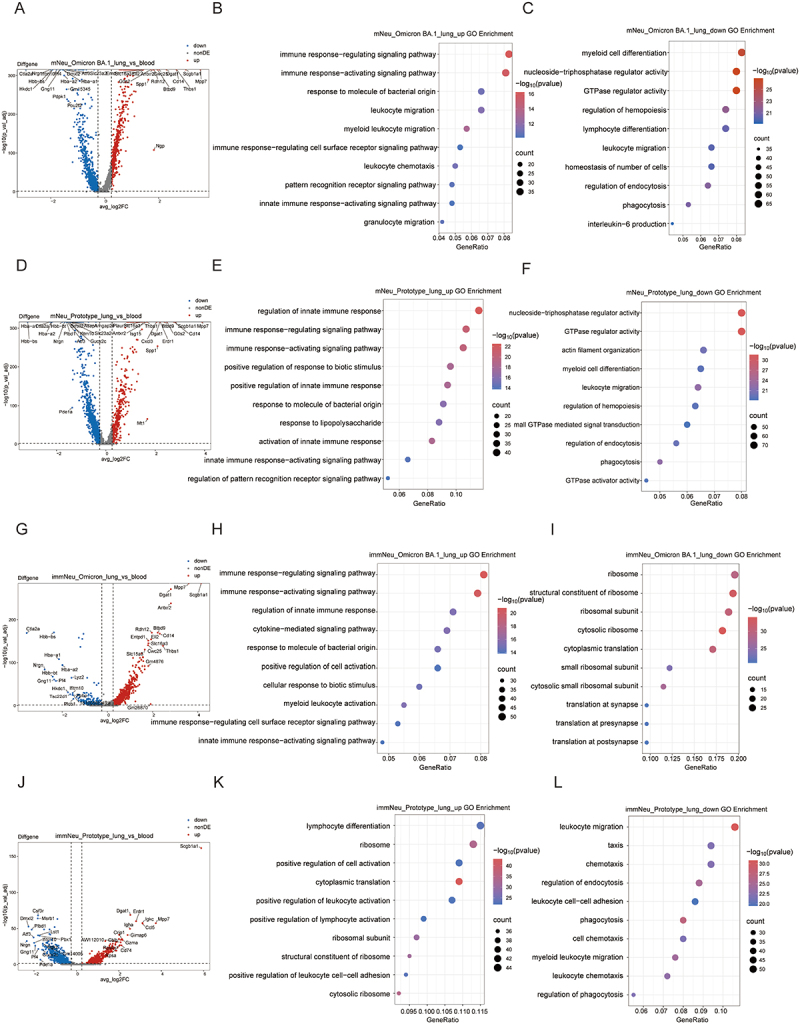
Comparsion of signaling pathways alteration in mNeu and imNeu between lung and blood. (A) the volcano plot of DEGs in mNeu between Omicron BA.1-infected lung and blood (|logfc| > 0.25, adjusted *p* value < 0.05). (B-C) Dotplots of top 10 GO terms in mNeu of upregulated/downregulated DEGs between Omicron BA.1-infected lung and blood. (D) the volcano plot of DEGs in mNeu between prototype-infected lung and blood (|logfc| > 0.25, adjusted *p* value < 0.05). (E-F) Dotplots of top 10 GO terms in mNeu of upregulated/downregulated DEGs between prototype-infected lung and blood. (G) the volcano plot of DEGs in immNeu between Omicron BA.1-infected lung and blood (|logfc| > 0.25, adjusted *p* value < 0.05). (|logfc| > 0.25, adjusted *p* value < 0.05). (H-I) Dotplots of top 10 GO terms in immNeu of upregulated/downregulated DEGs between Omicron BA.1-infected lung and blood. (J) the volcano plot of DEGs in immNeu between prototype-infected lung and blood (|logfc| > 0.25, adjusted *p* value < 0.05). (|logfc| > 0.25, adjusted *p* value < 0.05). (K-L) Dotplots of top 10 GO terms in immNeu of upregulated/downregulated DEGs between prototype-infected lung and blood.

Neutrophils originate in the bone marrow, are released into the peripheral blood upon maturation, and migrate from the PB to function in tissues when damage occurs [31]. Following a comparison of the functional enrichment of neutrophils between the two strains of infection, an analysis was conducted to examine the gene transcriptional alterations that occur in neutrophil subpopulations as they migrate from the PB to lung tissue. In the Omicron BA.1 group, mNeu was observed to upregulate genes such as *CD14*, *Thbs1*, and *Scgb1a1* in the lungs and downregulate genes such as *Hbb-bs*, *Ifitm10*, and *Hba-a1* when compared to peripheral blood (Figure 7(A)), and various immune response regulation and activation signaling pathways were upregulated (Figure 7(B) and S10(A)). Additionally, there was a trend toward upregulation of leukocyte chemotaxis and migration signaling pathways (Figure 7(B)). Conversely, downregulation of myeloid cell differentiation, GTPase regulation signaling pathways, and other signaling pathways were observed in mNeu from the lung compared to PB (Figure 7(C) and S10(B)). Meanwhile, the prototype group exhibited an increase in the expression of genes such as *CD14* and *Scgb1a1* in the mNeu of lungs (Figure 7(D)), which was accompanied by the activation of immune-related signaling pathways and downregulation of myeloid cell differentiation, GTPase regulation and other signaling pathways (Figure 7(E,F) and S10(C, D)). In the case of immNeu in the Omicron BA.1 group, the 15 most significantly upregulated and downregulated genes were found to be similar to those observed in mNeu, including *CD14*, *Mpp7*, *Scgb1a1*, *Btbd1* and *Thbs1* (Figure 7(G)). ImmNeu in the lungs also upregulated signaling pathways involved in the regulation and activation of the immune response compared to PB (Figure 7(H) and S10(E)). Furthermore, immNeu upregulated cytokine-mediated signaling pathway and downregulated ribosome-related signaling pathways in the lungs (Figure 7(H,I) and S10(E,F)). In contrast to the Omicron BA.1 group, immNeu in the lungs of the prototype group upregulated *CD74* expression compared to PB (Figure 7(J)). In addition, immNeu in the lungs of the prototype group positively regulated leukocyte and lymphocyte activation while downregulating chemotactic and migration pathways (Figure 7(K,L) and S10(G,H)).

### Functional analysis of hyperinflammatory monocytes and macrophages in prototype and Omicron BA.1

**Figure 8.**
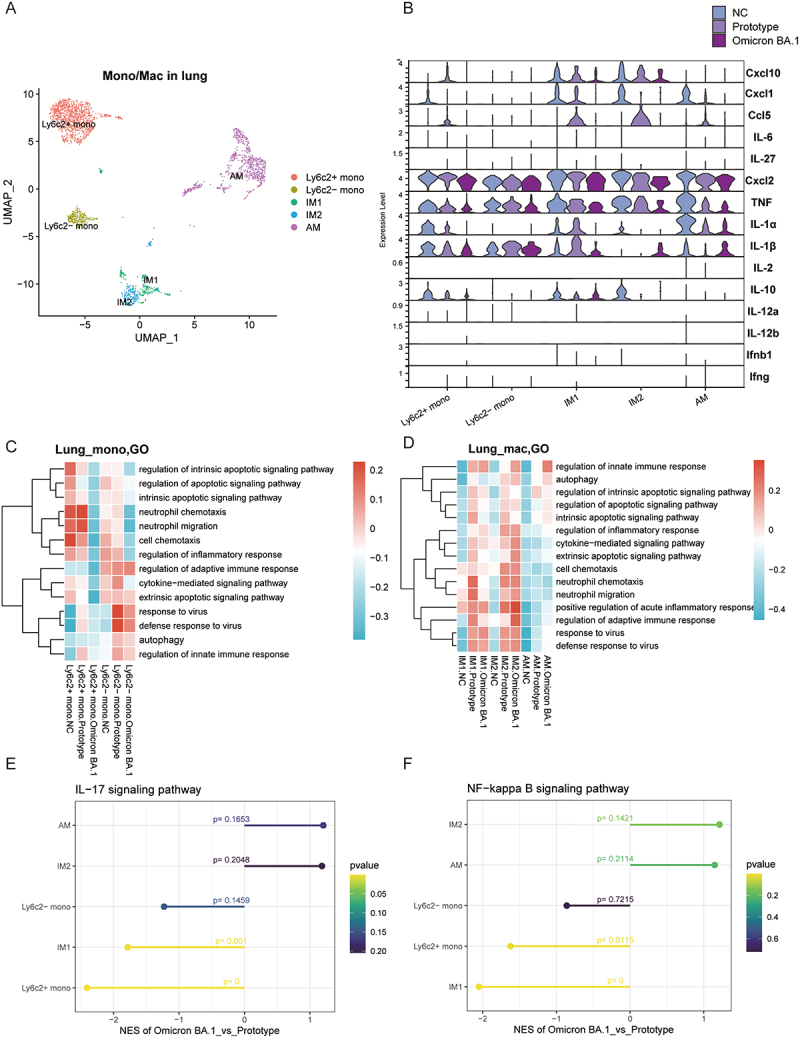
The functional monocytes and macrophages subsets in lung of prototype and Omicron BA.1 infected mice. (A) UMAP analysis of annotated monocytes and macrophages subsets in the lungs. (B) The violin diagram of expression in the genes related to inflammation. (C) Heatmap of GSVA scores with candidate interested GO terms among NC, prototype or Omicron BA.1 infection conditions in monocytes subsets in the lungs. (D) Heatmap of GSVA scores with candidate interested GO terms among NC, prototype or Omicron BA.1 infection conditions in macrophages subsets in the lungs. (E) GSEA of IL-17 signaling pathways in each monocyte and macrophage subset in the lungs. (F) GSEA of NF-kappa B signaling pathway in each monocyte and macrophage subset in the lungs.

Given the more pronounced inflammatory damage observed in the lungs of the prototype infection group, we examined the roles of monocytes and macrophages in the pathogenesis of this process. Five distinct types of monocytes and macrophages (Ly6c2^+^ monocytes, Ly6c2^−^ monocytes, IM1s, IM2s, and AMs) were identified in the lungs of mice (Figure 8(A)). The results of the mouse experiment showed that after knockout of monocytes and macrophages, the mice died earlier (Figure 5(A,B)). The expression of cytokines by monocytes and macrophages in each group was quantified, revealing diminished levels of *Cxcl10*, *Cxcl1*, *Ccl5*, *Cxcl2*, *Tnf*, *IL-1α*, and *IL-1β* in Ly6c2^+^ monocytes, IM1s, and AMs in the Omicron BA.1 group relative to the prototype group (Figure 8(B)). Additionally, the GSVA heatmap revealed that the Omicron BA.1 group exhibited downregulation of cell chemotaxis, cytokine-mediated signaling, and response to virus pathways in Ly6c2^+^ monocytes within the lungs and blood (Figure 8(C) and S11A,B). In contrast, these signaling pathways were upregulated in the lungs of the prototype group (Figure 8(C)). The Ly6c2^−^ monocyte population demonstrated an increase in immune-related signaling pathways within the lungs of both infected groups and the blood of the BA.1 group (Figure 8(C) and S11C,D). Moreover, GO-annotated modules revealed that the upregulation of positive regulation of acute inflammatory response, regulation of adaptive immune response, and response to virus pathways were observed in both infection groups in IM1 and IM2 (Figure 8(D)). The Omicron BA.1 infection resulted in a notable reduction in the expression of IL-17 and NF-κB signaling pathways in Ly6c2^+^ monocytes and IMs when compared to the prototype infection (Figure 8(E,F)). A similar trend was observed in Ly6c2^+^ monocytes in peripheral blood within the Omicron BA.1 group (S11E, F).

## Discussion

The present study characterized the single-cell landscape of the lungs and blood infected by the SARS-CoV-2 prototype or Omicron BA.1 variant in a mouse model during the late stages of infection. A variety of reduced lung tissue cells were identified, with capillary endothelial cells exhibiting the greatest number of DEGs in the infected groups compared to the NC group. The common characteristics of the SARS-CoV-2 prototype and Omicron BA.1 include the upregulation of the chemokine signaling pathway and IL-17 signaling pathway in capillary endothelial cells. Considering the impact of the divergence between the two strains, our findings indicated that the prototype strain exhibited upregulation of the oxidative stress pathway in comparison to the Omicron BA.1 strain. In the context of innate immunity, we conducted an in-depth analysis of immature and mature neutrophils, revealing that the two neutrophil subsets demonstrate a tendency to upregulate the genes associated with neutrophil functions in the lungs. Immature neutrophils in lungs upregulated wound healing pathway in Omicron BA.1 infection, compared to the prototype strain. The neutrophil subsets also upregulated the pathways related to the immune response in lungs, compared with blood. Moreover, Ly6c2^+^ monocytes and IM1 also exhibited downregulation of IL-17 and NF-κB signaling pathways in Omicron BA.1 infection.

The most pronounced transcriptional changes occurred in capillary endothelial cells, a key site of viral entry and immunopathology. Following stimulation of an inflammatory response, endothelial cells are capable of modulating the activity of both homeostatic and immune cells through a complex interaction of adhesion molecules, chemokines, and signaling pathways [32]. Both strains commonly upregulated chemokine and IL-17 signaling pathways in these cells (e.g. *Gnaq, Lyn, Rock2, Ccl5, Cxcl2, Vav3, Elmo1*), suggesting shared mechanisms for immune cell recruitment that contribute to lung inflammation. A comparative analysis of lung tissue differential genes between the two infection groups revealed that epithelial cell migration signaling pathways were upregulated in the lungs of the SARS-CoV-2 prototype infection group. According to a previous report, AT2s migrate after injury and exert the ability to complement AT1s [33]. This suggests that the damage to alveolar epithelial cells caused by prototype infection may have been more severe in the late stage of infection, to the extent that the migration signaling pathway of AT2s was upregulated. Crucially, prototype infection upregulated the oxidative stress regulator *Txnip* in lung cells compared to Omicron BA.1. Overexpression of *Txnip* has been demonstrated to result in the elevated production of ROS, which in turn promotes oxidative stress [34]. This finding strongly suggests enhanced oxidative stress contributes to the more severe lung injury characteristic of prototype infection.

Our analysis provides significant new insights into the role of neutrophils in SARS-CoV-2 pathogenicity. Our findings underscore the particularity of immature neutrophils in lung tissue infected with SARS-CoV-2 prototype. While both neutrophil subsets (immature and mature) showed increased activation signatures in the lung compared to blood, immature neutrophils exhibited starkly divergent profiles between strains. The high expression of genes related to neutrophil function, including *Mmp8*, *S100a8*, *S100a9*, and neutrophil granules, as well as the upregulation of pro-inflammatory and antiviral signaling pathways in the lungs infected with the prototype strain as compared with Omicron BA.1 indicate that it may contribute to more severe lung injury. This finding aligns with clinical observations linking immature neutrophil hyperactivation to severe COVID-19 [15, 17, 35–37]. Furthermore, the observation that immature neutrophils in Omicron BA.1-infected lungs exhibit upregulated signaling pathways for wound healing suggests that this neutrophil subset may possess a distinctive reparative capacity. This finding may offer a novel perspective on the mechanism of pathologic attenuation following BA.1 infection. However, additional evidence is required to substantiate this hypothesis. We also documented distinct alterations in gene expression profiles as neutrophils migrated from blood to lung tissue. In tumor studies, CD74^+^ neutrophils have been observed to exhibit antigen-presenting functions that induce T-cell antigen-specific responses [38]. Therefore, it is also possible that the elevation of *CD74* expression in the lung in comparison to the peripheral blood could be indicative of specific functions for immature neutrophils, including the presentation of antigens, although further studies are required to confirm this hypothesis.

Similarly, key innate immune mononuclear phagocytes exhibited dampened inflammatory responses in Omicron BA.1 infection. Monocytes and macrophages are thought to be the main triggers of cytokine storms in patients with severe COVID-19 [39]. Ly6c2^+^ monocytes, also referred to as classic monocytes, are believed to differentiate into macrophages with pro-inflammatory properties, whereas Ly6c2^−^ monocytes typically differentiate into anti-inflammatory macrophages. AMs serve as the primary defenders of the alveoli and airways, whereas lung IMs primarily protect the vasculature and lung interstitium [40].IM1 displays a pro-inflammatory phenotype and expresses IL-1β, whereas IM2 highly expresses CD163 and primarily exerts anti-inflammatory functions [41]. Our findings revealed the expression of chemokines, TNF, IL-1α, and other cytokines in pro-inflammatory macrophages and classical monocytes in the Omicron BA.1 group was observed to be lower than that of the prototype group. It appears that pro-inflammatory classical monocytes and IM1s play a primary role in prototype infection, activating downstream inflammatory signaling pathways, including the IL-17 and NF-κB pathways. IL-17 is a pro-inflammatory cytokine that is commonly expressed. Previous research has demonstrated that IL-17 levels are elevated in individuals with severe cases of SARS-CoV-2 infection compared to those with mild symptoms [42]. Other literature has demonstrated that the SARS-CoV-2 open reading frame 8 (ORF8) has the capacity to bind to the IL-17 receptor and activate the IL-17 signaling pathway, thereby triggering a robust inflammatory response [43]. Activation of the NF-κB signaling pathway has also been observed in SARS-CoV-2 [44]. It has been demonstrated that NF-κB activation may trigger the production of downstream pro-inflammatory cytokines (e.g. IL-6 and IL-8), which initiate a cytokine storm and lead to ARDS [45]. Our data indicated that the chemotactic and pro-inflammatory signaling pathway genes of monocytes and macrophages, which are responsible for pro-inflammatory functions, are expressed at lower levels in the Omicron strain than the prototype strain. This phenomenon may be associated with a decrease in the severity of inflammation, as observed by lung histopathology.

Despite the provision of a comparison of the changes in cellular and immune responses in the blood and lung tissue caused by SARS-CoV-2 prototype and Omicron BA.1 strain at the single-cell level, the present study is not without limitations. Firstly, the use of an hACE2 transgenic mouse model, while essential for studying SARS-CoV-2 pathogenesis, may not fully recapitulate all aspects of human COVID-19, particularly regarding specific immune cell subsets or complex systemic responses. Secondly, the present analysis was focused on significant late-time points following infection, and the incorporation of early dynamic changes in the host response could offer further insights. Finally, this study focused on innate immunity and lung tissue cells, and interactions with adaptive immunity require further investigation.

In summary, the primary indication of SARS-CoV-2-induced immunological damage to lung tissue was gene alterations in capillary endothelial cells. Gene expression and regulatory processes of signaling pathways within neutrophil subpopulations and monocytes/macrophages were also investigated. Furthermore, the underlying reasons for the different histopathologic damage to the lungs and the severity of the disease exhibited by the two SARS-CoV-2 strains of infection (that is, the prototype and the Omicron BA.1 variant) were explored, leading to new insights into disease progression.

## Supplementary Material

S11.tif

S5.tif

S2.tif

S8.tif

S7.tif

S3.tif

S1.tif

S9.tif

S4.tif

S6.tif

Supplementary figure legends.docx

S10.tif

## Data Availability

The raw sequence data reported in this paper have been deposited and openly available in the Genome Sequence Archive (GSA) with the accession number CRA019030 at https://ngdc.cncb.ac.cn/gsa. The other data that support the findings of this study are openly available in Mendeley Data with DOI:10.17632/9y93mjff4b.1 at https://data.mendeley.com/datasets/9y93mjff4b/1.

## ARRIVE guidelines statement

The authors confirm we have adhered to ARRIVE guidelines.
