# Supplementary figures and images for: Single-cell transcriptomic atlas of blood and lung from mice infected with SARS-CoV-2 revealing distinct virulence characteristics between prototype and Omicron BA.1 strain
Source: Virulence. 2025 Aug 28;16(1):2548931. doi: 10.1080/21505594.2025.2548931 (PMC12396132; doi:10.1080/21505594.2025.2548931)

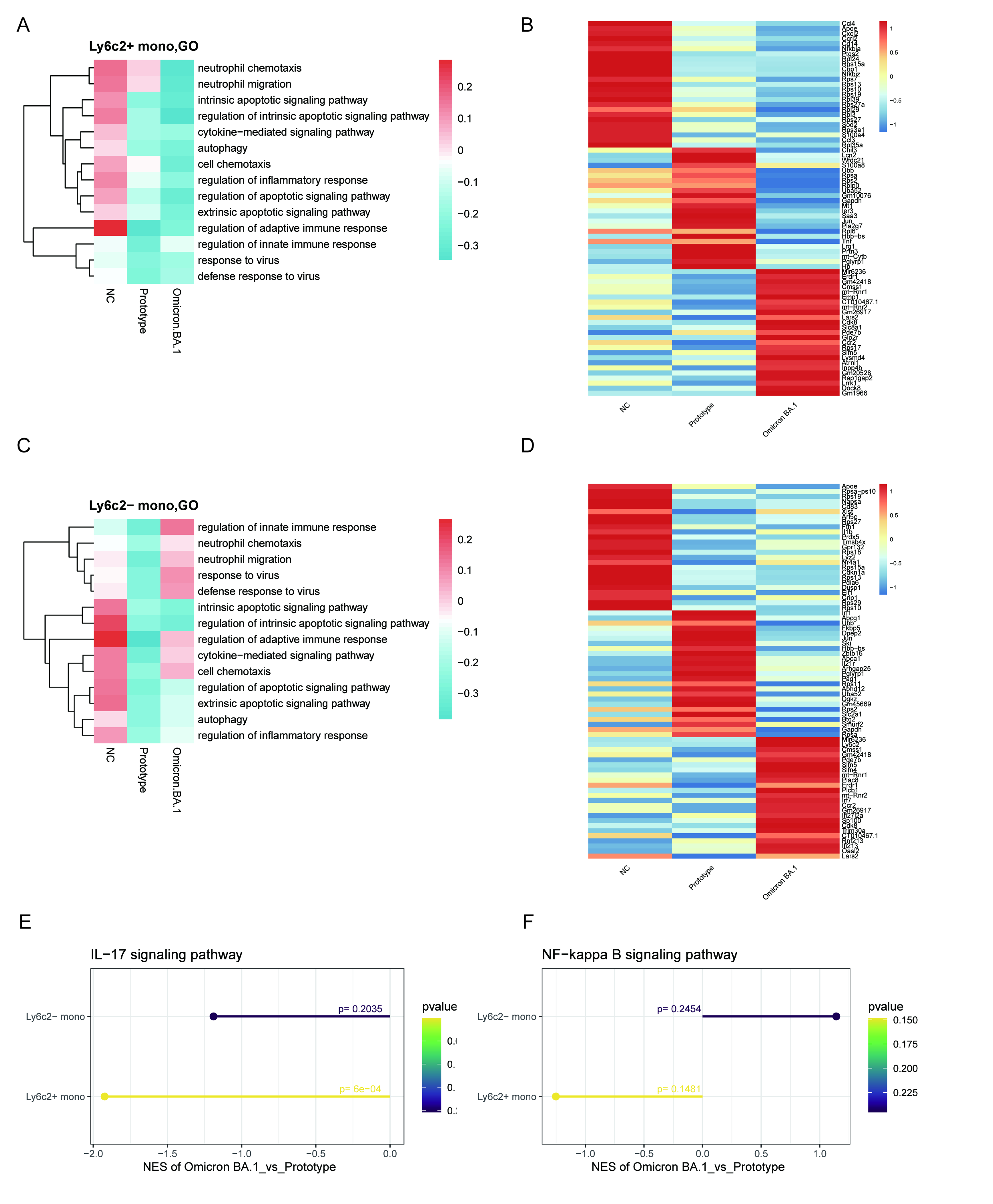

Supplement: S11.tif [file KVIR_A_2548931_SM1850.tif]

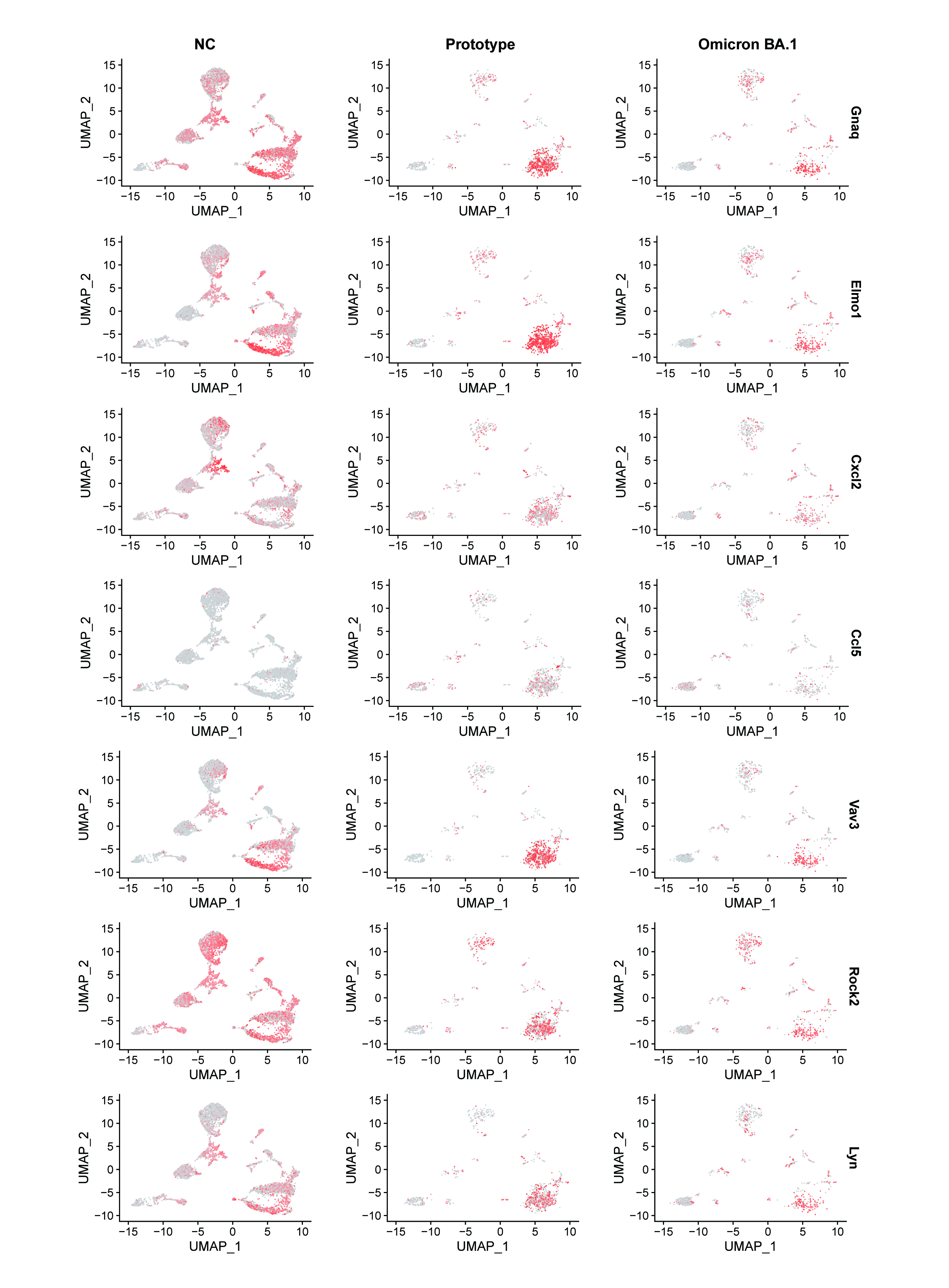

Supplement: S5.tif [file KVIR_A_2548931_SM1849.tif]

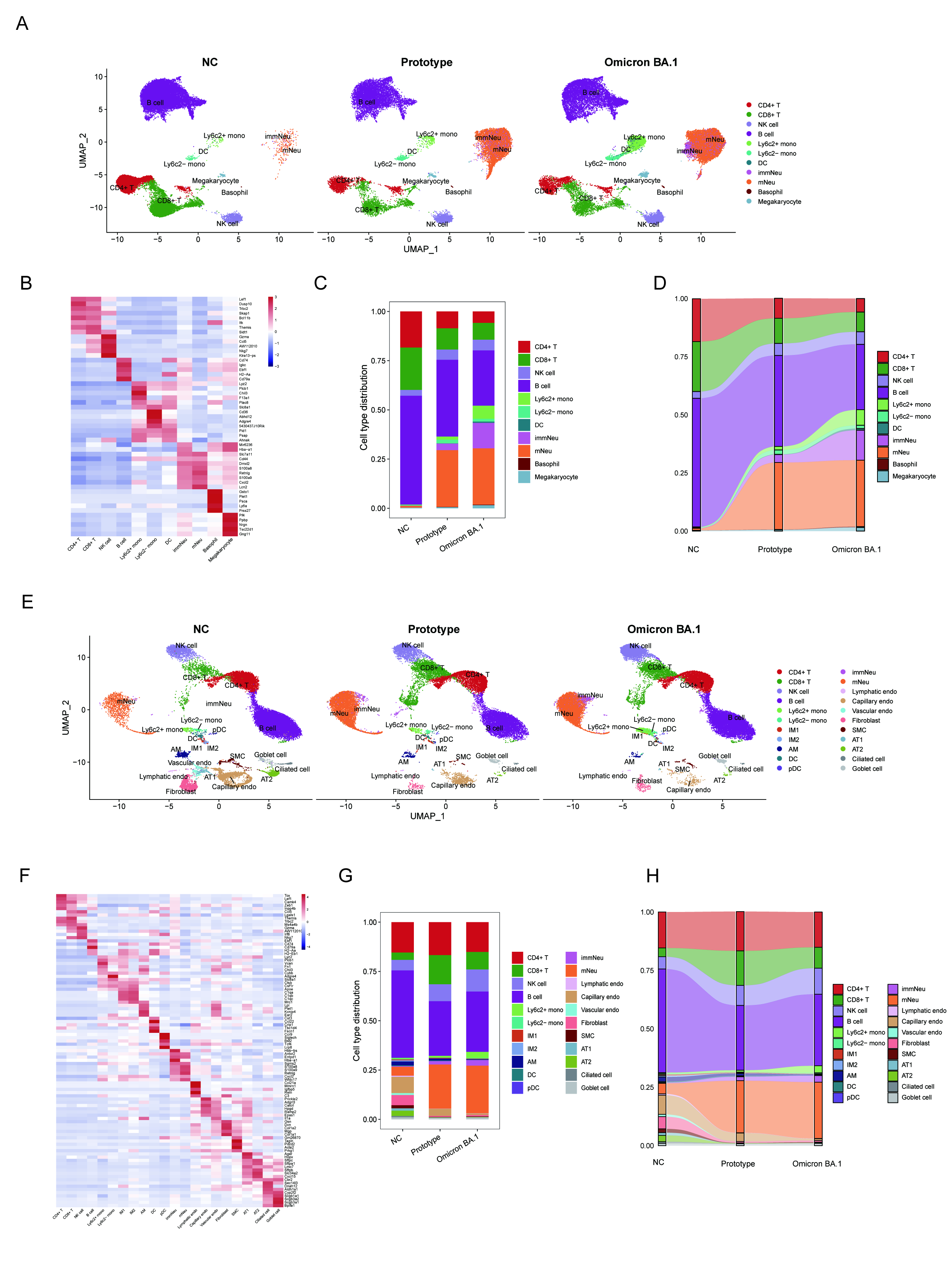

Supplement: S2.tif [file KVIR_A_2548931_SM1848.tif]

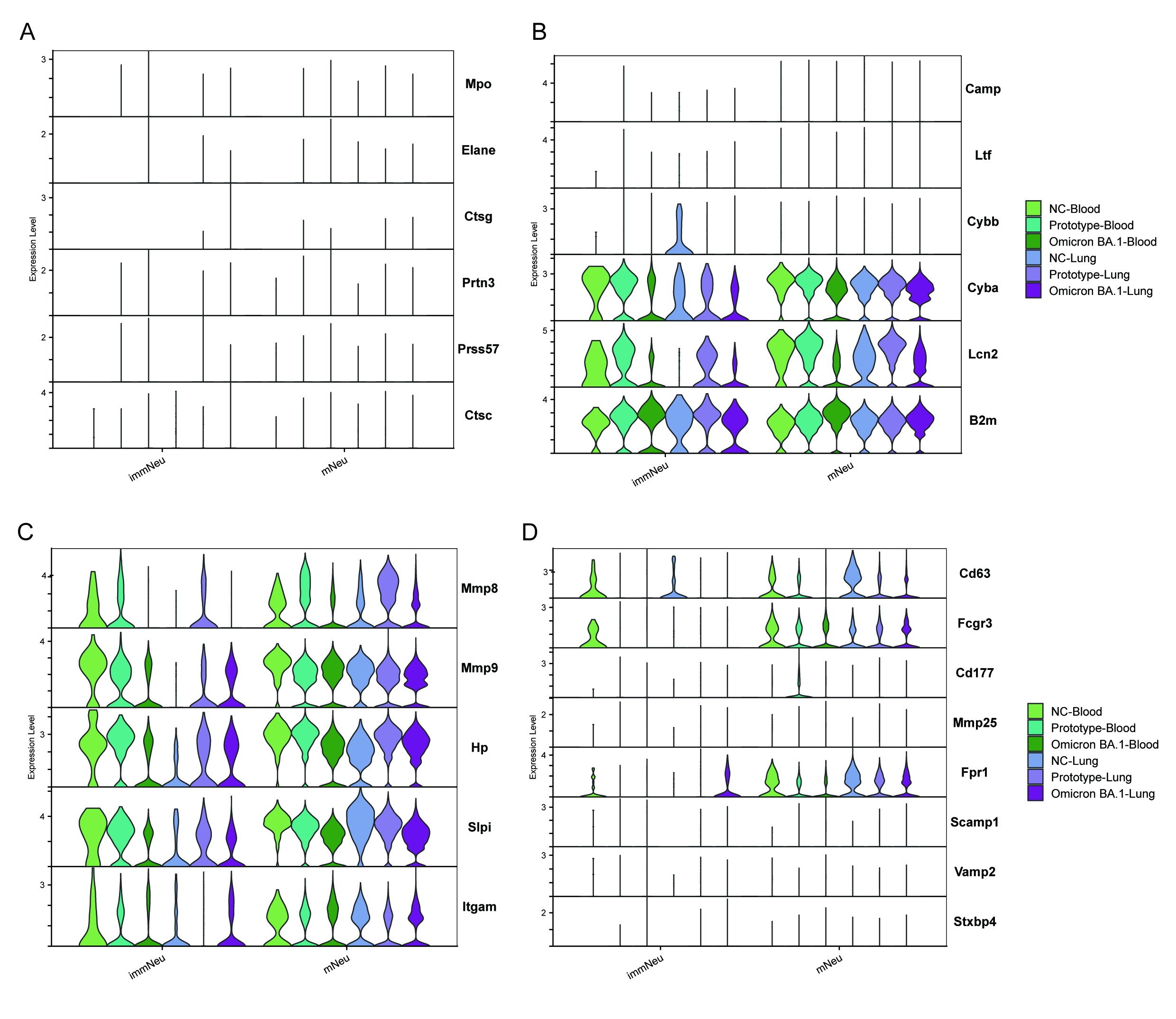

Supplement: S8.tif [file KVIR_A_2548931_SM1847.tif]

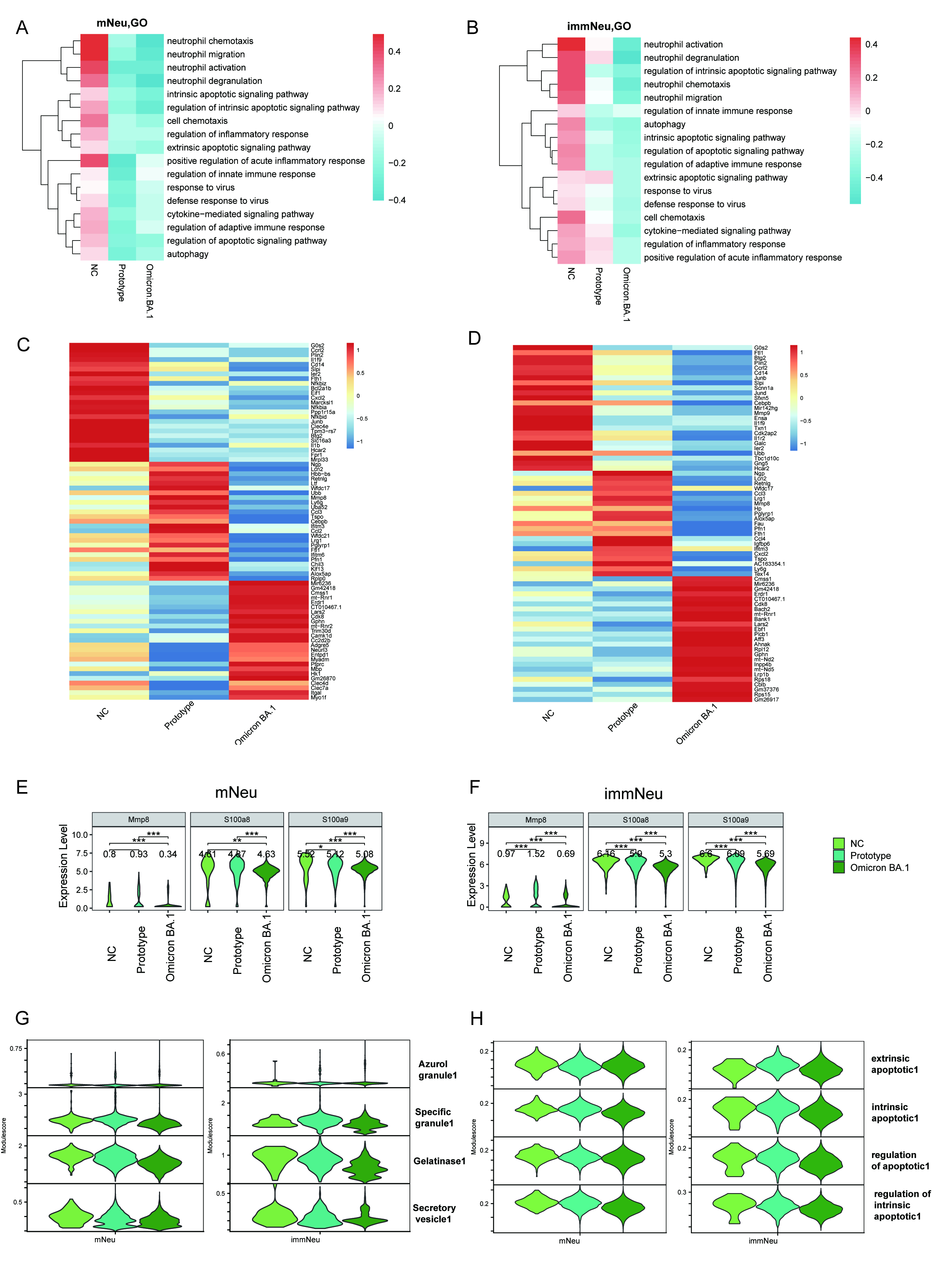

Supplement: S7.tif [file KVIR_A_2548931_SM1846.tif]

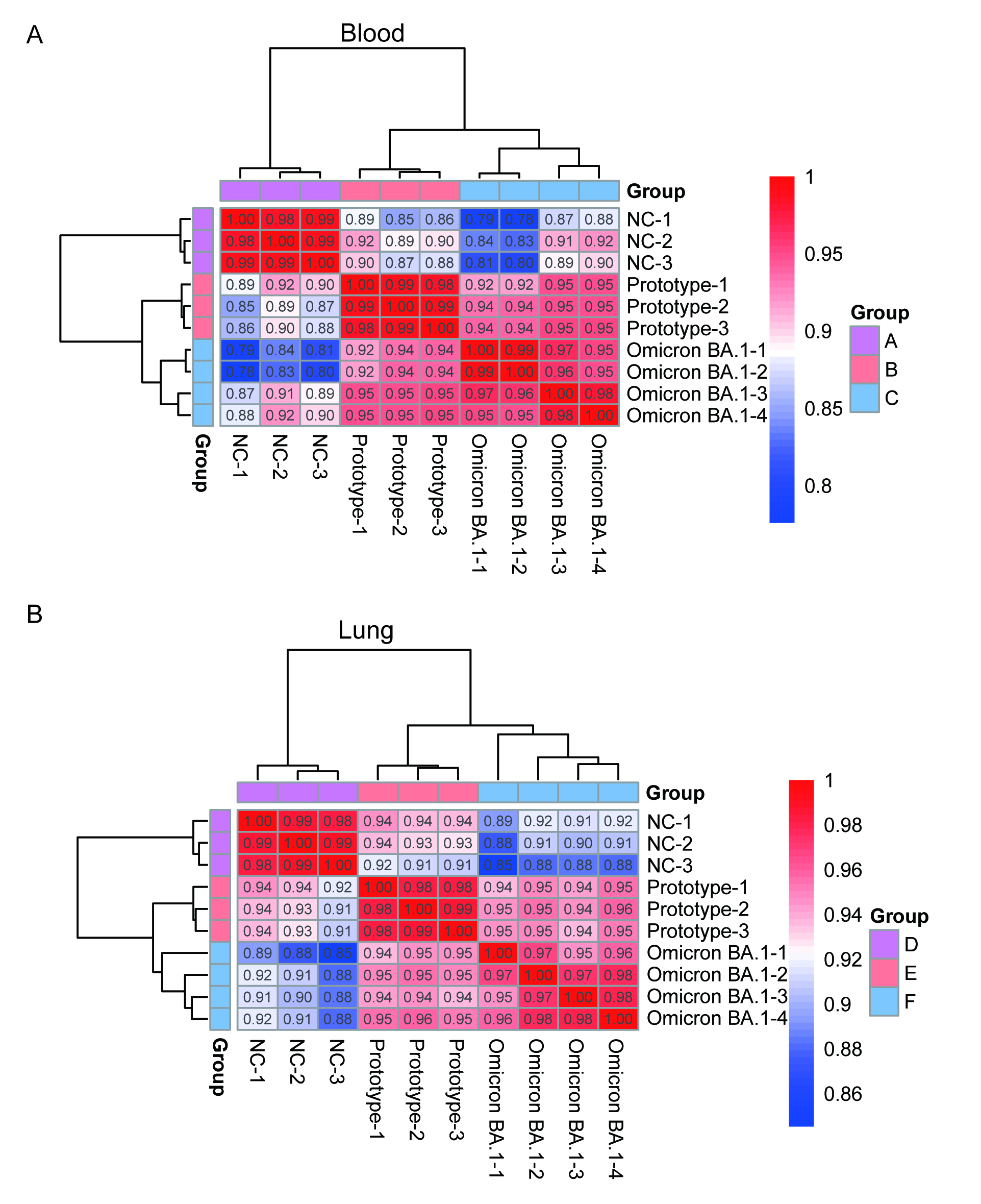

Supplement: S1.tif [file KVIR_A_2548931_SM1844.tif]

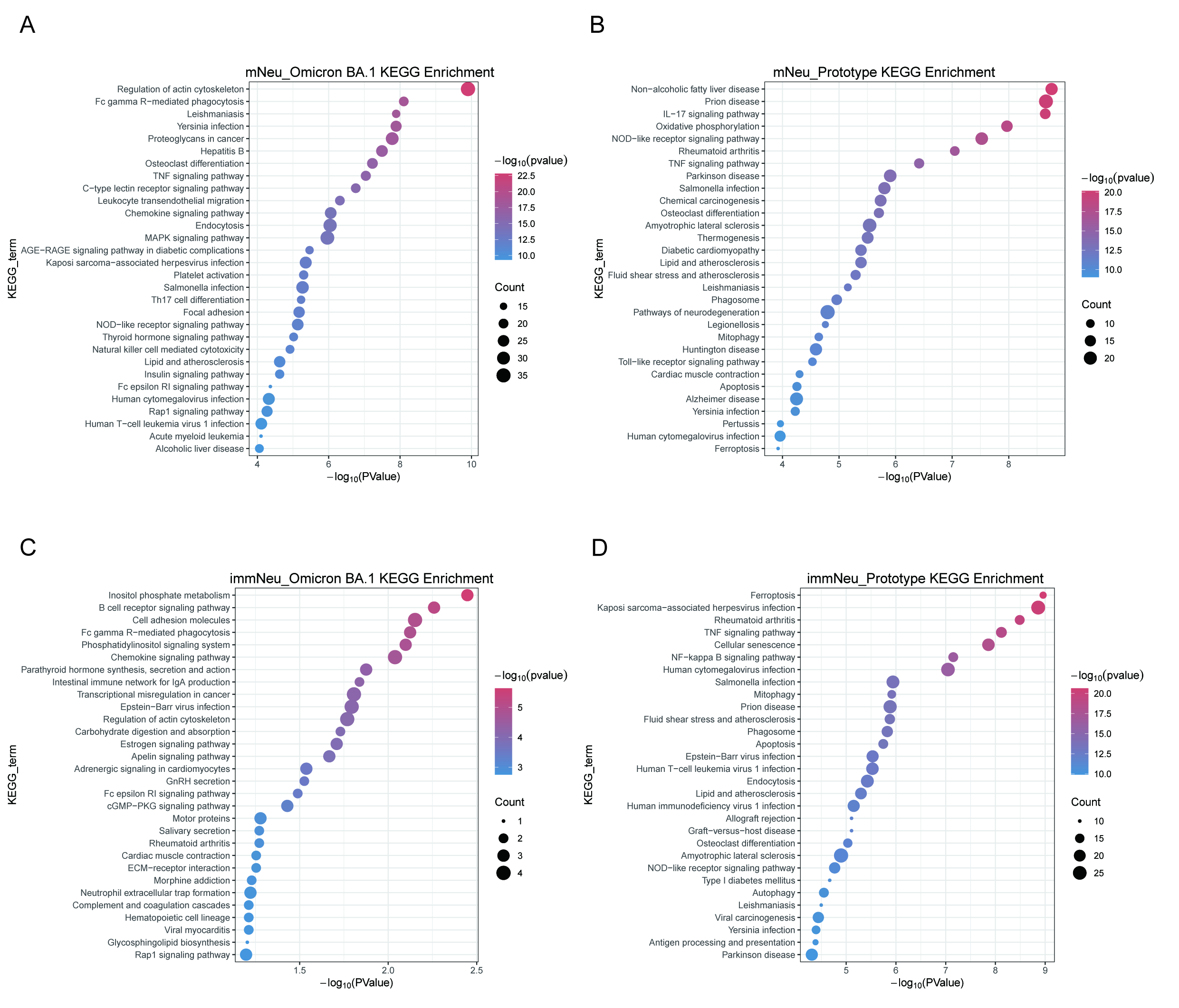

Supplement: S9.tif [file KVIR_A_2548931_SM1843.tif]

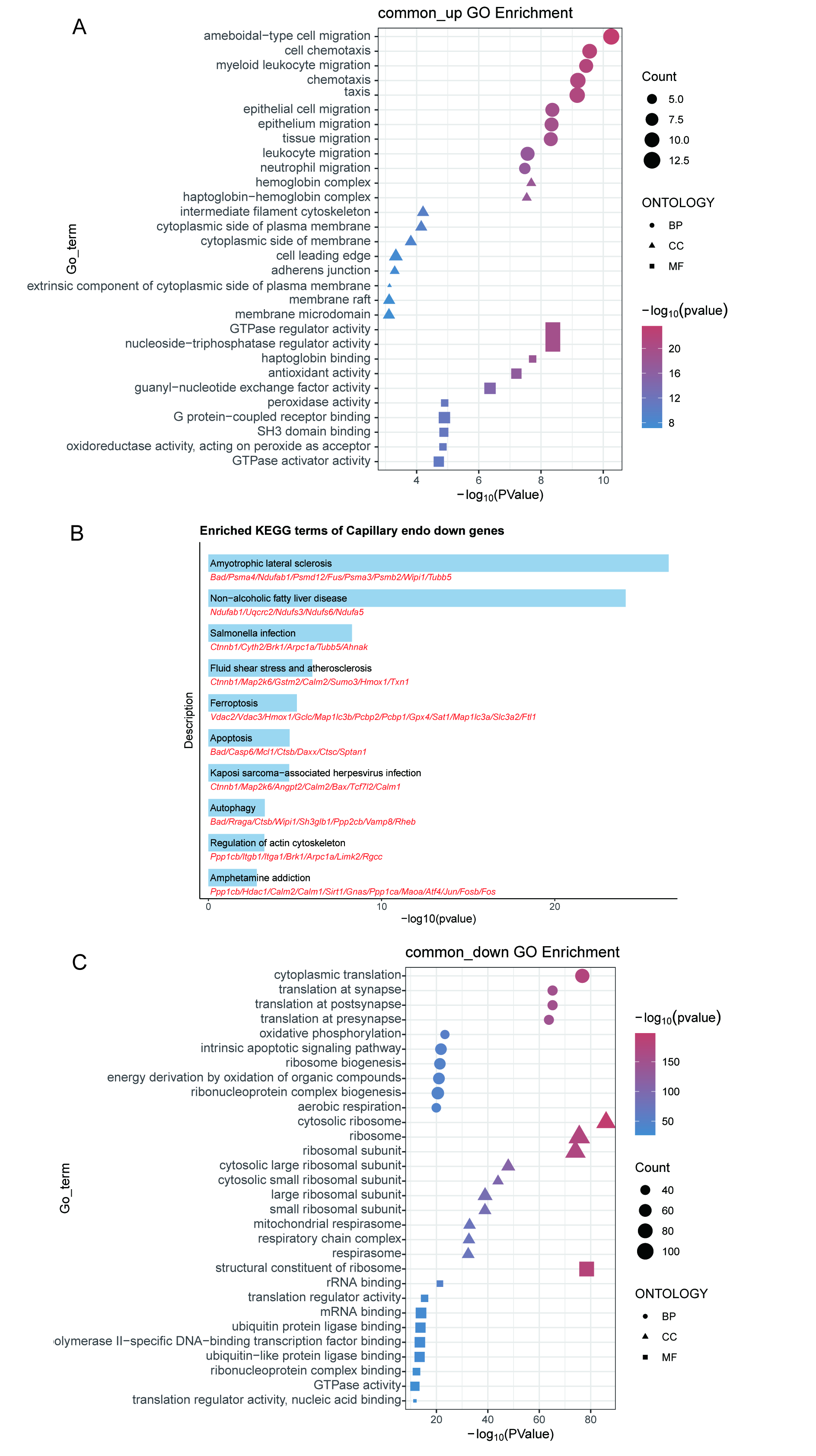

Supplement: S4.tif [file KVIR_A_2548931_SM1842.tif]

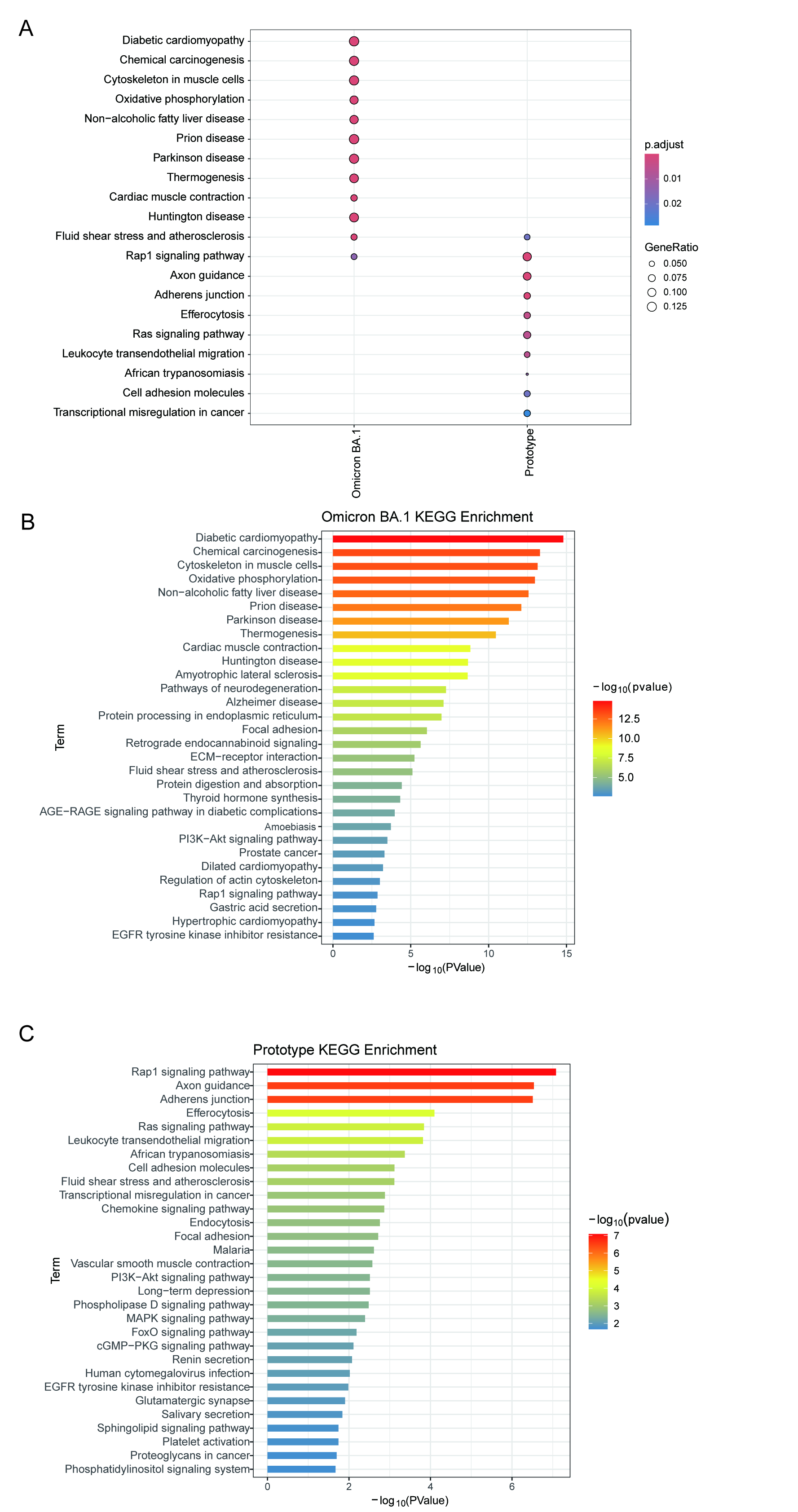

Supplement: S6.tif [file KVIR_A_2548931_SM1841.tif]

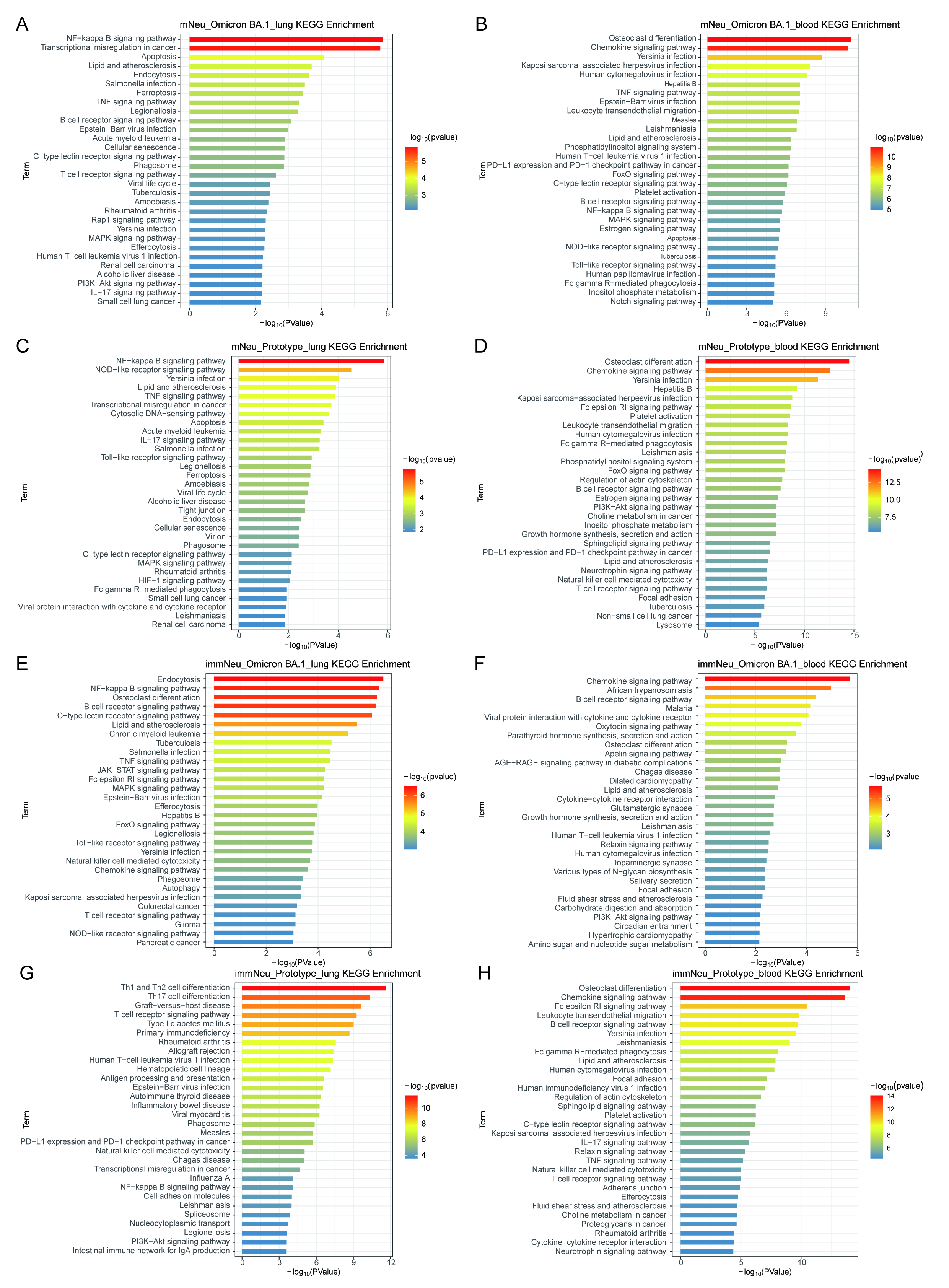

Supplement: S10.tif [file KVIR_A_2548931_SM1839.tif]
