# Supplementary material for: Single-cell transcriptomic atlas of blood and lung from mice infected with SARS-CoV-2 revealing distinct virulence characteristics between prototype and Omicron BA.1 strain
Source: Virulence. 2025 Aug 28;16(1):2548931. doi: 10.1080/21505594.2025.2548931 (PMC12396132; doi:10.1080/21505594.2025.2548931)
Supplement: Supplementary figure legends.docx [file KVIR_A_2548931_SM1840.docx]

**Figure S1 Hierachical clustering using the Spearman correlation coefficient of a normalized transcriptome between blood/lung samples.** (A) Blood. (B) Lung.

**Figure S2** **Distribution of cell types in blood and lung of mice challenged with SARS-CoV-2 prototype or Omicron BA.1.**

(A) UMAP analysis of annotated cell types in peripheral blood (PB) leukocytes in NC (negative control), Prototype and Omicron BA.1 conditions. (B) Heatmap of top 5 marker genes in blood cell types. (C) Barplot of blood cell types distribution in NC, Prototype and Omicron BA.1 conditions. (D) Sankey diagrams plot of blood cell types distribution in NC, Prototype and Omicron BA.1 conditions. (E) UMAP analysis of annotated cell types in lung with differential infection conditions (NC, Prototype or Omicron BA.1 infection). (F) Heatmap of top 5 marker genes in lungs. (G) Bar plot of lung cell types distribution in NC, Prototype and Omicron BA.1 infection conditions. (H) Sankey diagrams plot of lung cell types distribution in NC, Prototype and Omicron BA.1 infection conditions.

**Figure S3 Comparison of gene expression in lungs between infection groups and NC group**

(A) UMAP analysis of annotated lung tissue cell types in lungs with differential conditions (NC, Prototype and Omicron BA.1). (B-C) The volcano plot of up-regulated and down-regulated DEGs of each cell type in the lungs in Prototype vs NC (B), and Omicron vs NC (C) condition (|logFC| > 0.25, adjusted *P* value < 0.05). The annotated genes in the left volcano plot were the top 5 DEGs in each cell type, and the annotated genes in the right volcano plot were the top 10 up-regulated and down-regulated DEGs.

**Figure S4 Functional enrichment analysis of common DEGs in pulmonary capillary endothelial cells.**

(A) GO functional enrichment dot plot obtained from 74 co-upregulated genes.

(B) KEGG functional enrichment bar plot obtained from 1,213 co-downregulated genes.

(C) GO functional enrichment dot plot obtained from 1,213 co-downregulated genes.

**Figure S5** **Expression of genes in chemokine signaling pathway in lungs.**

The expression of *Gnaq*, *Elm1*, *Cxcl2*, *Ccl5*, *Vav3*, *Rock2*, *Lyn* genes in the 3 groups of lung tissue cells UMAP. The degree of gene expression was positively correlated with the degree of redness.

**Figure S6** **KEGG functional enrichment of DEGs in lungs between prototype and Omicron BA.1 infection.**

(A) Dotplots of top10 enrichment KEGG terms with differential genes between Omicron BA.1 and Prototype conditions in lungs. (B) Bar chart of top 30 enrichment KEGG terms in upregulated DEGs in Omincron BA.1 group. (C) Barchart of top 30 enrichment KEGG terms in upregulated DEGs of Prototype group.

**Figure S7 Analysis of neutrophils subsets in blood of prototype and Omicron BA.1 infected mice.**

(A) Heatmap of GSVA scores with candidate interested GO terms among NC, Prototype and Omicron BA.1 conditions in mNeu in the blood. (B) Heatmap of GSVA scores with candidate interested GO terms among NC, Prototype and Omicron BA.1 conditions in immNeu in the blood. (C) Heatmaps of top 25 differential genes among NC, Prototype and Omicron BA.1 conditions with mNeu in the blood. (D) Heatmaps of top 25 differential genes among NC, Prototype and Omicron BA.1 conditions with immNeu in the blood. (E) Expression comparison of *Mmp8*, *S100a8* and *S100a9* genes among NC, Prototype and Omicron BA.1 in mNeu of blood. (F) Expression comparison of *Mmp8*, *S100a8* and *S100a9* genes among NC, Prototype and Omicron BA.1 in immNeu of lungs. (G) The violin diagram showed the score of neutrophil granule gene in immNeu and mNeu of blood. (H) The violin diagram drawn the expression of apoptotic pathways in immNeu and mNeu of blood. *: P<0.05; **: P<0.01; ***: P<0.001.

**Figure S8 ModuleScore of neutrophil granule-related genes**

(A-D) ModuleScore of the primary (azurophilic) granules (A), secondary (specific) granules (B), teritary (gelatinase) granules (C), and secretory vesicles (D) signature gene of mature and immature neutrophils in the mice infected with SARS-CoV-2 prototype or Omicron BA.1.

**Figure S9 KEGG functional enrichment of DEGs in mNeu and immNeu between prototype and Omicron BA.1 infected lungs**

(A) Dotplots of top 30 enrichment KEGG terms with upregulated DEGs in mNeu of Omicron BA.1 infected lungs. (B) Dotplots of top 30 enrichment KEGG terms with upregulated DEGs in mNeu of prototype infected lungs. (C) Dotplots of top30 enrichment KEGG terms with upregulated DEGs in immNeu of Omicron BA.1 infected lungs. (D) Dotplots of top30 enrichment KEGG terms with upregulated DEGs in immNeu of prototype infected lungs.

**Figure S10 KEGG functional enrichment of DEGs in mNeu and immNeu between lung and blood**

(A) Bar chart of top 30 enrichment KEGG terms with upregulated DEGs in mNeu of Omicron BA.1 infected lungs. (B) Bar chart of top 30 enrichment KEGG terms with upregulated DEGs in mNeu of Omicron BA.1 infected blood. (C) Bar chart of top 30 enrichment KEGG terms with upregulated DEGs in mNeu of prototype infected lungs. (D) Bar chart of top 30 enrichment KEGG terms with upregulated DEGs in mNeu of prototype infected blood. (E) Bar chart of top 30 enrichment KEGG terms with upregulated DEGs in immNeu of Omicron BA.1 infected lungs. (F) Bar chart of top 30 enrichment KEGG terms with upregulated DEGs in immNeu of Omicron BA.1 infected blood. (G) Bar chart of top 30 enrichment KEGG terms with upregulated DEGs in immNeu of prototype infected lungs. (H) Bar chart of top30 enrichment KEGG terms with upregulated DEGs in immNeu of prototype infected blood.

**Figure S11 The functional monocytes subsets in blood of prototype and Omicron BA.1 infected mice.**

(A) Heatmap of GSVA scores with candidate interested GO terms among NC, Prototype and Omicron BA.1 conditions in Ly6c2+ monocytes in the blood. (B) Heatmap of GSVA scores with candidate interested GO terms among NC, Prototype and Omicron BA.1 conditions in Ly6c2+ monocytes in the blood. (C) Heatmaps of top 25 differential genes among NC, Prototype and Omicron BA.1 conditions with Ly6c2- monocyte in the blood. (D) Heatmaps of top 25 differential genes among NC, Prototype and Omicron BA.1 conditions with Ly6c2- monocyte in the blood. (E) GSEA of IL-17 signaling pathways in monocytes subsets in the blood. (F) GSEA of NF-kappa B signaling pathway in each monocytes subset in the blood.
